# Synthesis and biological evaluation of benzyloxymethyl-substituted imidazo[1,2-a]pyridines as apoptosis-inducing anticancer agents through modulation of BCL-2/Bax signaling and caspase-3 activation

**DOI:** 10.1007/s00210-026-05638-6

**Published:** 2026-06-26

**Authors:** Ege Arzuk, İbrahim Pinar, Burak Kuzu

**Affiliations:** 1 Department of Pharmaceutical Toxicology, Faculty of Pharmacy, Ege University, İzmir, 35040 Türkiye; 2 Department of Pharmaceutical Chemistry, Faculty of Pharmacy, Van Yuzuncu Yil University, Van, 65080 Türkiye

**Keywords:** Anticancer agents, Apoptosis, Bax, BCL-2, Caspase-3, Imidazopyridine

## Abstract

A novel series of benzyloxymethyl-substituted imidazo[1,2-a]pyridine derivatives (**IPO1-IPO14**) was designed, synthesized, and evaluated as potential anticancer agents. The compounds were synthesized via an efficient multistep strategy with satisfactory yields and structurally characterized by comprehensive ^1^H, ^13^C NMR, and LC-TOF analyses. Antiproliferative activities were evaluated against a panel of human cancer cell lines, including HepG2, A549, PC-3, MDA-MB-231, and MCF-7 cells; A549 non-small cell lung cancer and MDA-MB-231 triple-negative breast cancer cells showed the highest sensitivity. Among the tested compounds, **IPO8**, **IPO9**, **IPO10**, **IPO11**, and **IPO13** demonstrated the strongest antiproliferative activity. **IPO9** emerged as the most potent derivative, displaying significant cytotoxicity against A549 (IC_50_ = 3.84 µM) and MDA-MB-231 cells (IC_50_ = 9.69 µM), together with favorable selectivity profiles (SI = 23.02 and 15.31, respectively). Structure–activity relationship analysis revealed that para-alkoxy substitution enhanced antiproliferative potency and selectivity, whereas bulky aromatic or strongly electron-withdrawing substituents reduced biological activity. Mechanistic investigations suggested that the lead derivatives induced mitochondria-mediated intrinsic apoptosis through suppression of BCL-2, induction of Bax expression, elevation of the Bax/BCL-2 ratio, disruption of mitochondrial membrane potential, and activation of caspase-3. Among the tested compounds, **IPO9** consistently produced the strongest pro-apoptotic effects and, in MDA-MB-231 cells, exhibited more pronounced modulation of several apoptosis-related markers than the positive control doxorubicin. Further molecular docking studies revealed favorable binding interactions of **IPO9** within the BCL-2 binding pocket, providing computational support for the *in vitro* findings.

**Graphical abstract:**

**Figure:**
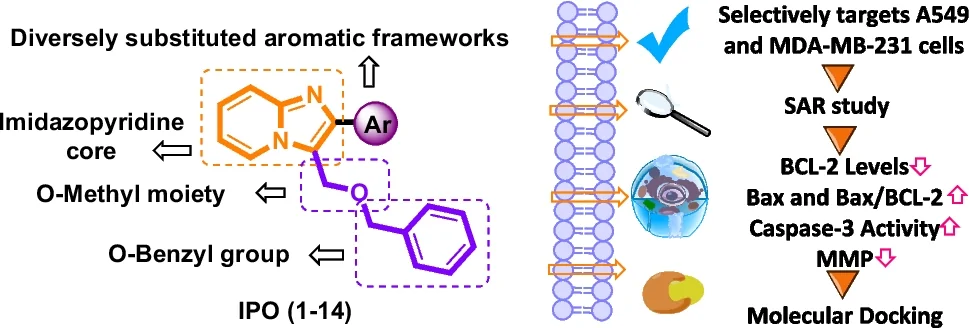

**Supplementary Information:**

The online version contains supplementary material available at https://doi.org/10.1007/s00210-026-05638-6.

## Introduction

Cancer remains a major global health burden, characterized by uncontrolled cell proliferation and the ability of malignant cells to evade programmed cell death (apoptosis), one of the fundamental hallmarks of cancer (Guo et al. 2026). Restoration of apoptotic signaling has become an important strategy in anticancer drug development, as it enables the selective elimination of cancer cells while minimizing damage to normal tissues (Min and Lee 2022). The mitochondria-mediated intrinsic apoptotic pathway plays a central role in regulating cell fate and is frequently dysregulated in cancer. The intrinsic apoptotic pathway is tightly regulated by members of the BCL-2 protein family, which govern mitochondrial outer membrane integrity and cellular susceptibility to apoptotic stimuli (Fernald and Kurokawa 2013). Under physiological conditions, anti-apoptotic proteins such as BCL-2 preserve mitochondrial integrity and prevent apoptosis. However, in response to cellular stress, pro-apoptotic proteins, including Bax and Bak, become activated, leading to mitochondrial outer membrane permeabilization and the release of cytochrome c into the cytosol (Kaloni et al. 2023). This event triggers apoptosome formation and initiates a cascade of caspase activation that ultimately culminates in activation of caspase-3 and apoptotic cell death (Tian et al. 2024). Conversely, overexpression of anti-apoptotic BCL-2 proteins inhibits Bax/Bak activation and prevents mitochondrial outer membrane permeabilization, thereby suppressing caspase activation and promoting tumor survival (Fernald and Kurokawa 2013). Accordingly, the balance between anti-apoptotic BCL-2 and pro-apoptotic Bax has emerged as a critical determinant of cellular susceptibility to intrinsic apoptosis, and disruption of this balance is frequently associated with cancer progression and therapeutic resistance. Therefore, therapeutic strategies aimed at restoring apoptotic signaling through modulation of the BCL-2/Bax axis and downstream caspase pathways have gained considerable attention in cancer drug development (Hussar 2022; Tian et al. 2024). Consequently, substantial efforts have been directed toward the discovery of structurally diverse small molecules capable of efficiently modulating apoptosis-related signaling, particularly the BCL-2/Bax axis (Cui et al. 2019; Huang et al. 2018, 2019).

**Fig. 1:**
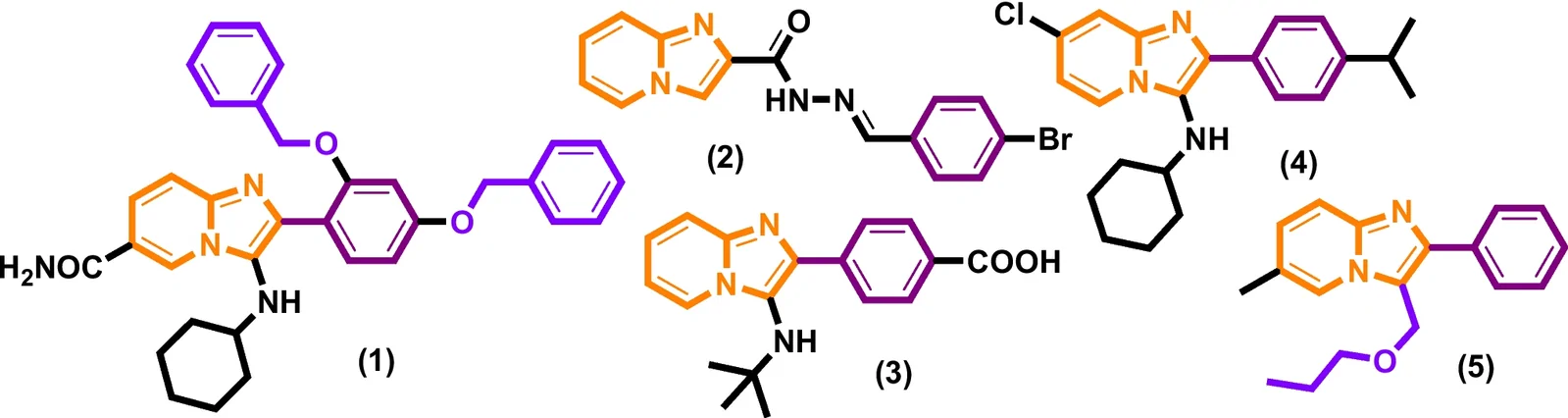
Selected literature-reported imidazo[1,2-a]pyridine derivatives (1–5) exhibiting apoptosis-inducing anticancer activity

Heterocyclic scaffolds have become prominent in anticancer drug discovery studies due to their structural diversity and their ability to form specific interactions with biological macromolecules (Al-Jumaili et al. 2025). Notably, the imidazo[1,2-a]pyridine core has emerged as a privileged pharmacophore, exhibiting a wide range of biological activities, including significant anticancer potential (Ferreira et al. 2026). For instance, amino-substituted imidazopyridine derivatives have been investigated for their antiproliferative effects on HT-29 and Caco-2 cell lines. Among these, the benzyl-substituted compound **1** exhibited pronounced cytotoxicity (IC_50_ values of 9.2 μM and 17.38 μM, respectively) and induced apoptosis via a caspase-mediated mechanism (Dahan-Farkas et al. 2011). Building on this structural framework, carbohydrazide-derived imidazopyridine analogs (**2**) were rationally designed and exhibited marked cytotoxic effects against a range of cancer cell lines, including MCF-7, HCT-116, and A549, while displaying minimal toxicity to normal cells (Firouzi et al. 2024). Similarly, 2-aryl-substituted imidazo[1,2-a]pyridin-3-amine analogs have emerged as potent inhibitors of Topo IIα, exhibiting significant anticancer efficacy in breast cancer cell models. Notably, compound **3**, displayed the highest activity, achieving an IC_50_ of 15 μM against MCF-7 cells (Baviskar et al. 2011). Continued structural refinement yielded compound **4**, which exhibited markedly improved cytotoxic potency in the same cell line (IC_50_ = 1 μM) (Yadav et al. 2022). In addition, leveraging these precedents, the design of a 3-O-methyl imidazopyridine derivative (compound **5**) led to a compound that exhibited cytotoxic activity against multiple cancer cell lines, including HCT-116, A549, MiaPaca-2, and MCF-7, with IC_50_ values of 47.2, 44.2, 31.1, and 20.1 μM, respectively (Fig. 1) (Singh et al. 2022). Taken together, these precedents underscore the therapeutic promise of imidazo[1,2-a]pyridine derivatives and provide a strong scientific rationale for further structural refinements to optimize their mitochondria-mediated pro-apoptotic efficacy.

Importantly, structural alterations of the imidazopyridine core have been demonstrated to significantly modulate anticancer properties. Strategic substitutions at various positions allow for precise adjustment of electronic characteristics, distribution, lipophilicity, and binding affinity to molecular targets (Kuzu 2024). For instance, functionalization with aryl, heteroaryl, and methylene-linked substituents has been shown to enhance cytotoxic potency and promote apoptosis, predominantly through activation of caspase-3 and modulation of the PI3K/AKT signaling pathway (Singh et al. 2022). These findings underscore the importance of rational substituent selection in optimizing biological activity. Accordingly, a benzyloxymethyl group was introduced as a structure-guided modification to enhance anticancer efficacy. Medicinal chemistry analyses reveal that the benzyloxymethyl moiety confers several key advantages: (*i*) increased lipophilicity, facilitating improved cellular membrane permeability, (*ii*) the presence of an aromatic benzyl unit enabling π-π stacking interactions within the hydrophobic binding domain, and (*iii*) a flexible methylene linker, allowing optimal pharmacophore orientation. Collectively, these structural characteristics are recognized for their capacity to enhance ligand–protein interactions and have been extensively utilized in the development of apoptosis-modulating agents (Souers et al. 2013). Although benzyloxymethyl ether functionalities may undergo CYP-mediated O-dealkylation, this moiety was selected for its favorable contributions to lipophilicity, membrane permeability, and hydrophobic interactions with biological targets. Furthermore, preliminary *in silico* ADMET analyses indicated acceptable drug-likeness and pharmacokinetic characteristics for the evaluated compounds. Therefore, the benzyloxymethyl group was considered a suitable structural feature for exploring structure–activity relationships, while its potential metabolic susceptibility remains a subject for future lead-optimization studies.

**Fig. 2:**
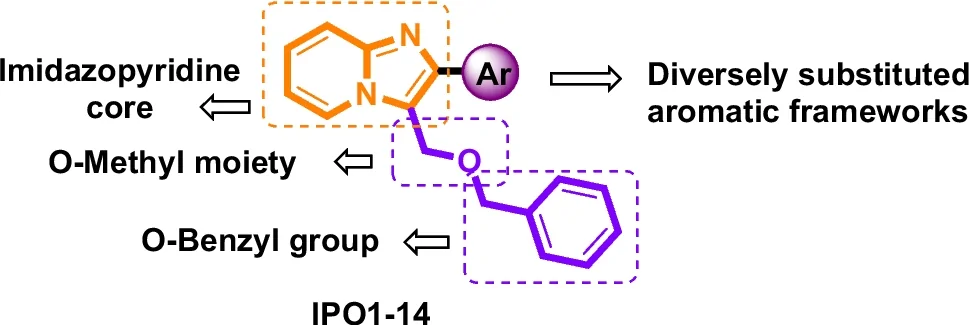
Rational design of benzyloxymethyl-substituted imidazo[1,2-a]pyridine derivatives

Driven by these insights, we designed a novel series of benzyloxymethyl-substituted imidazo[1,2-a]pyridine derivatives, strategically integrating a privileged heterocyclic scaffold with a targeted lipophilic substituent to modulate apoptosis-associated pathways (Fig. 2). This rational design was predicated on the hypothesis that such structural features would enhance binding affinity toward anti-apoptotic proteins, notably Bax, BCL-2, thereby promoting downstream caspase-3 activation.

In this study, we present the synthesis and thorough biological characterization of these derivatives. Structure–activity relationship (SAR) analysis was conducted to elucidate the molecular features governing anticancer activity. In addition, the most potent compounds were subjected to mechanistic investigations, including assessment of their effects on BCL-2 and Bax protein levels, the Bax/BCL-2 ratio, mitochondrial membrane potential, and caspase-3 activation in cancer cells, thereby providing mechanistic insight into their pro-apoptotic potential.

## Results and discussion

### Chemistry

**Fig. 3:**
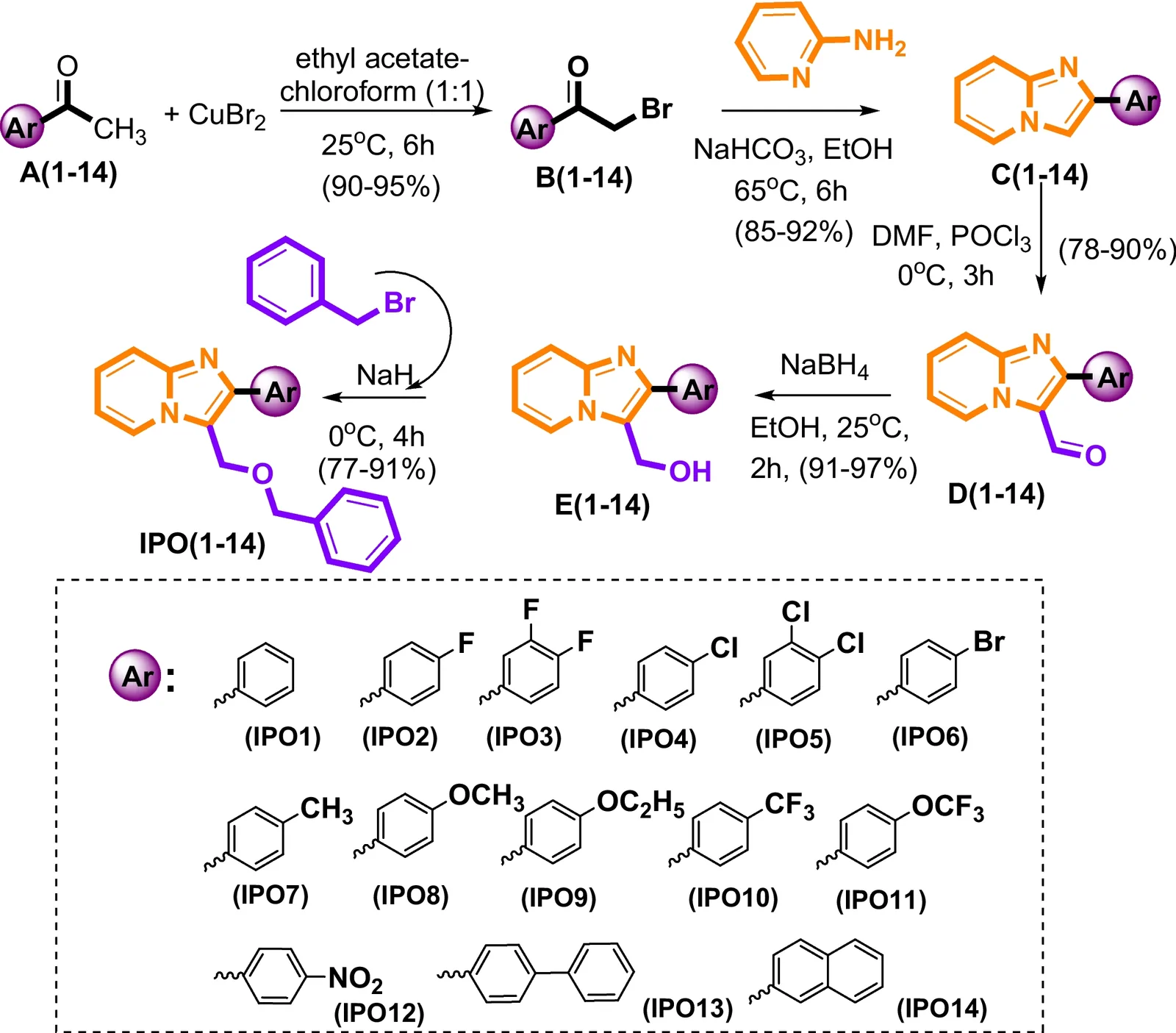
General synthesis scheme of the target compounds **(IPO1-14)** with stepwise yields and Ar substituent definitions included as a legend

The synthetic strategy devised for the preparation of the target heterocyclic hybrids (**1–14**) is depicted in Fig. 3 and features a concise, modular, and operationally straightforward sequence with consistently high stepwise yields of 77%−97% throughout the route. In the initial step, acetyl-substituted aromatic precursors **A(1–14)** undergo regioselective α-bromination in the presence of CuBr_2_ to afford the corresponding α-haloketone intermediates **B(1–14)** in 90–95% yield. This transformation efficiently activates the benzylic position, rendering it amenable to subsequent annulation. In the second stage, intermediates **B(1–14**) react with 2-aminopyridine to furnish aryl-substituted imidazo[1,2-a]pyridine derivatives **C(1–14)** via a cyclocondensation process in 85–92% yield. This step proceeds in excellent yields and effectively transfers the structural diversity of the Ar substituents (**1–14**) onto the heterocyclic core, enabling systematic modulation of electronic and steric parameters across the series. Subsequently, the imidazopyridine intermediates **C(1–14)** are subjected to Vilsmeier-Haack formylation (DMF/POCl_3_, 0 °C), thereby regioselectively installing an aldehyde group on the heteroaromatic framework, yielding **D(1–14)** in 78–90% yield. This electrophilic formylation step is both efficient and chemoselective, providing a versatile synthetic handle for downstream transformations. In the following step, the aldehyde group in **D(1–14)** is smoothly reduced using NaBH_4_ in ethanol at ambient temperature (25 °C), affording the corresponding hydroxymethyl-substituted derivatives **E(1–14)** in 91–97% yield. The mild reaction conditions ensure high chemoselectivity while preserving the integrity of the imidazopyridine scaffold. Finally, O-benzylation of intermediates **E(1–14)** with benzyl bromide in the presence of NaH (0 °C, 4 h) delivers the desired target compounds **(1–14)** in good to excellent yields 77–91% yield. The introduction of the O-benzyl moiety not only enhances lipophilicity but also contributes an additional aromatic domain that may facilitate favorable intermolecular interactions in biological environments. Overall, this well-orchestrated synthetic route from **A(1–14)** to **(IPO1-14)** provides a robust and versatile platform for generating structurally diverse imidazopyridine-based hybrids, thereby enabling systematic exploration of structure–activity relationships through rational variation of the Ar substituents.

The target compounds **(IPO1-14)** were isolated in good to excellent yields (77–91%) as analytically pure solids or viscous liquids, with consistent melting points and R_f_ values (0.125–0.30, ethyl acetate/n-hexane 1:3), supporting their efficient formation and clean purification. Their structures were unambiguously established by ^1^H- and ^13^C-NMR spectroscopy, which consistently confirmed the successful construction of the imidazo[1,2-a]pyridine core and the installation of the O-benzyl functionality. In the ^1^H-NMR spectra, all derivatives displayed two diagnostic singlets at δ ≈ 4.85–4.98 ppm and δ ≈ 4.55–4.65 ppm, attributable to the benzylic -OCH_2_- group and the adjacent methylene linker, respectively. The aromatic region (δ ≈ 6.7–8.2 ppm) exhibited well-resolved multiplets corresponding to the fused heteroaromatic system and the aryl substituents, with substituent-dependent splitting patterns clearly evident.

The influence of electronic effects is particularly reflected in both proton and carbon spectra. Fluorinated derivatives such as **IPO2** and **IPO3** showed characteristic coupling patterns, including pronounced ^13^C-^19^F coupling (e.g., δ ≈ 162.7 ppm, d, *J* ≈ 247 Hz for **IPO2**), whereas strongly electron-withdrawing groups such as -CF_3_ (**IPO10**) and -NO_2_ (**IPO14**) induced notable downfield shifts in the aromatic signals. Conversely, electron-donating substituents, as in **IPO8** (-OCH_3_) and **IPO9** (-OC_2_H_5_), gave rise to additional aliphatic resonances (δ ≈ 55.3 ppm and δ ≈ 63.4/14.9 ppm, respectively) and subtle shielding effects within the aromatic region. In the ^13^C NMR spectra, the expected aromatic carbon signals (δ ≈ 112–160 ppm) were accompanied by distinct resonances for the benzylic and methylene carbons at δ ≈ 72 ppm (-OCH_2_-) and δ ≈ 60 ppm (-CH_2_-), respectively. The spectral data were confirmed by ^1^H, ^13^C NMR, LC-TOF/MS analyses, and the corresponding spectra are available in the Supplementary Material (Figures S1-S42). Altogether, the spectroscopic data are fully consistent with the proposed structures and confirm the successful synthesis of a structurally diverse series of imidazopyridine-based hybrids, systematically functionalized with electronically differentiated aryl groups.

### Biological evaluation

#### Cytotoxicity studies by MTT assay

The antiproliferative activities of the synthesized benzyloxymethyl-substituted imidazo[1,2-a]pyridine derivatives (**IPO1-14**) were systematically evaluated against a panel of human cancer cell lines, including HepG2, A549, PC-3, MDA-MB-231, and MCF-7 cells. The corresponding IC_50_ values are summarized in Table 1, with doxorubicin (Dox) used as the reference compound.

**Table 1:** In vitro cytotoxic activity (IC_50_, µM) of **IPO1-14** derivatives across human cancer and non-tumorigenic cell lines

Compounds	IC_50_ (µM)						
	HepG2	A549	PC-3	MDA-MB-231	MCF-7	MCF-10A	NIH/3T3
IPO1	92.15 ± 0.42	100.21 ± 0.09	125.18 ± 0.51	83.18 ± 0.27	75.62 ± 0.26	172.15 ± 1.26	169.82 ± 0.36
IPO2	73.85 ± 0.19	89.46 ± 0.21	47.15 ± 0.19	69.62 ± 0.14	66.09 ± 0.21	135.26 ± 0.81	156.84 ± 0.69
IPO3	63.47 ± 0.58	69.05 ± 1.13	42.08 ± 0.73	36.54 ± 0.63	26.54 ± 0.94	142.15 ± 0.19	148.94 ± 1.12
IPO4	69.1 ± 0.29	68.40 ± 0.49	29.17 ± 0.62	29.04 ± 0.43	45.53 ± 0.97	152.21 ± 1.02	148.47 ± 0.39
IPO5	48.75 ± 0.19	36.48 ± 0.16	25.09 ± 1.28	34.29 ± 1.06	24.61 ± 0.51	144.8 ± 0.75	159.33 ± 0.64
IPO6	46.21 ± 0.81	37.21 ± 0.44	26.34 ± 1.53	36.51 ± 1.15	22.36 ± 0.61	142.36 ± 0.39	148.92 ± 1.21
IPO7	78.54 ± 0.16	88.63 ± 1.12	45.57 ± 0.87	69.41 ± 0.81	65.21 ± 1.09	162.31 ± 0.54	156.39 ± 1.09
IPO8	14.41 ± 0.64	**5.46 ± 0.52**	19.07 ± 1.09	**10.26 ± 1.03**	26.35 ± 0.98	152.06 ± 0.91	92.36 ± 1.12
IPO9	21.39 ± 0.29	**3.84 ± 0.62**	22.64 ± 1.12	**9.69 ± 0.19**	10.41 ± 1.04	148.32 ± 0.26	84.39 ± 0.36
IPO10	35.73 ± 0.43	**5.62 ± 0.94**	44.8 ± 1.08	**8.42 ± 0.76**	21.32 ± 0.16	121.18 ± 0.42	102.74 ± 0.49
IPO11	57.48 ± 1.09	**9.85 ± 1.01**	32.23 ± 0.39	**8.63 ± 0.29**	41.36 ± 0.19	136.42 ± 0.14	108.26 ± 1.06
IPO12	50.19 ± 0.83	13.08 ± 0.66	30.03 ± 1.21	25.84 ± 0.06	74.32 ± 0.69	145.18 ± 0.18	162.09 ± 1.11
IPO13	32.86 ± 0.67	**6.61 ± 0.95**	64.34 ± 1.52	**9.87 ± 0.64**	26.54 ± 0.25	153.09 ± 1.12	157.36 ± 0.94
IPO14	96.71 ± 0.29	97.25 ± 0.18	113.08 ± 1.08	89.47 ± 0.32	79.32 ± 0.61	142.5 ± 1.23	168.14 ± 0.67
Doxorubicin (reference drug)	1.19 ± 0.21	1.15 ± 0.18	1.12 ± 0.35	0.94 ± 0.13	0.62 ± 0.18	4.26 ± 0.12	3.35 ± 0.18

The **IPO** series exhibited marked structure-dependent differences in cytotoxic activity, with IC_50_ values ranging from low micromolar concentrations to > 100 µM depending on both structural features and cancer cell type. Overall, the derivatives displayed limited potency against HepG2 and PC-3 cells. In contrast, several compounds demonstrated pronounced antiproliferative activity against A549 and MDA-MB-231 cells, suggesting a cell-line-dependent cytotoxic profile. Notably, A549 cells consistently exhibited the highest sensitivity across the series, followed by MDA-MB-231 cells. Interestingly, MDA-MB-231 triple-negative breast cancer cells were generally more sensitive to the synthesized derivatives than MCF-7 estrogen receptor-positive breast cancer cells. Given the aggressive phenotype and limited targeted therapeutic options associated with triple-negative breast cancer (Raman et al. 2025), this enhanced sensitivity may be of particular therapeutic relevance. Among the tested cancer cell lines, such differential sensitivity may arise from variations in cellular uptake, apoptotic susceptibility, or oncogenic signaling pathways, as previously demonstrated in the imidazo[1,2-a]pyridine derivatives with pronounced antiproliferative and apoptosis-inducing effects (Altaher et al. 2022). In the present series, derivatives bearing para-alkoxy substituents exhibited enhanced activity, further suggesting that electron-donating alkoxy groups on the imidazo[1,2-a]pyridine scaffold may contribute to enhanced antiproliferative and pro-apoptotic activity. In contrast, bulky aromatic substituents or strongly electron-withdrawing groups such as nitro generally reduced cytotoxic potency. Compounds with IC_50_ values below 10 µM were selected for further mechanistic investigations.

The initial derivatives, **IPO1-4**, exhibited relatively weak antiproliferative activity, particularly against HepG2 and A549 cells, with IC_50_ values that predominantly exceeded 60 µM. **IPO1**, representing the unsubstituted scaffold, displayed the lowest activity and served as a reference compound for subsequent analogs. Moderate improvements were observed for **IPO3** and **IPO4**, particularly against MDA-MB-231 and PC-3 cells. **IPO5** and **IPO6** exhibited intermediate activity across most cell lines, whereas **IPO7** showed comparatively lower potency, indicating that its substitution pattern was less favorable for cytotoxic activity.

Among the synthesized derivatives, **IPO8** and **IPO9** emerged as the most potent compounds. **IPO9** demonstrated the strongest activity against A549 cells (IC_50_ = 3.84 ± 0.62 µM) and also exhibited pronounced cytotoxicity against MDA-MB-231 (IC_50_ = 9.69 ± 0.19 µM) and MCF-7 cells (IC_50_ = 10.41 ± 1.04 µM). The superior activity of **IPO9** may be associated with the favorable balance between lipophilicity and electronic properties conferred by the ethoxy substituent, which is in agreement with earlier medicinal chemistry studies demonstrating that electron-donating substituents on imidazo[1,2-a]pyridine scaffolds can enhance anticancer activity (Krishnamoorthy and Anaikutti 2023). Importantly, **IPO9** exhibited sub-10 µM activity against both A549 and MDA-MB-231 cells while maintaining favorable selectivity toward non-tumorigenic cells. Similarly, **IPO8** displayed strong activity against both A549 (IC_50_ = 5.46 ± 0.52 µM) and MDA-MB-231 (IC_50_ = 10.26 ± 1.03 µM) cells. Additional derivatives, including **IPO10**, **IPO11**, and **IPO13**, also demonstrated notable antiproliferative effects, particularly against A549 and MDA-MB-231 cells, with IC_50_ values predominantly below 10 µM. In contrast, **IPO14** exhibited low cytotoxicity across all tested cell lines, with IC_50_ values exceeding 79 µM, indicating limited antiproliferative activity. Collectively, **IPO8**, **IPO9**, **IPO10**, **IPO11**, and **IPO13** were identified as the most promising derivatives within the series.

To further evaluate the selectivity profiles of the synthesized compounds, selectivity indices (SI) were determined using non-tumorigenic MCF-10A and NIH/3T3 cells as controls (Table 2). The majority of active derivatives exhibited greater cytotoxicity toward malignant cells than toward non-tumorigenic cells. Notably, **IPO8-IPO13** exhibited the most favorable selectivity profiles against A549 and MDA-MB-231 cells. Among these, **IPO9** demonstrated particularly high selectivity, with SI values of 23.02 and 15.31 against A549 and MDA-MB-231 cells, respectively. **IPO13** displayed particularly high selectivity toward A549 cells despite exhibiting only moderate activity against other cancer cell lines, suggesting a more selective cytotoxic profile. Overall, the combination of potent antiproliferative activity and favorable selectivity profiles identified **IPO8**, **IPO9**, **IPO10**, **IPO11**, and **IPO13** as promising anticancer lead compounds for subsequent mechanistic evaluation.

**Table 2:** Selectivity indices (SI) of **IPO** derivatives were calculated using MCF-10A and NIH/3T3 as reference cell lines

Compounds	Selectivity Index (SI)				
	HepG2#	A549 #	PC-3#	MDA-MB-231 *	MCF-7*
IPO1	1.84	1.69	1.36	2.07	2.28
IPO2	2.12	1.75	3.33	1.94	2.05
IPO3	2.35	2.16	3.54	3.89	5.36
IPO4	2.15	2.17	5.09	5.24	3.34
IPO5	3.27	4.37	6.35	4.22	5.88
IPO6	3.22	4.00	5.65	3.90	6.37
IPO7	1.99	1.76	3.43	2.34	2.49
IPO8	5.72	15.08	4.32	14.82	5.77
IPO9	4.13	23.02	3.90	15.31	14.25
IPO10	2.88	18.28	2.29	4.77	5.68
IPO11	1.88	10.99	3.36	6.31	3.30
IPO12	3.23	12.39	5.40	5.62	1.95
IPO13	4.79	23.81	2.45	9.65	5.77
IPO14	1.74	1.73	1.49	1.59	1.80
Doxorubicin (reference drug)	2.82	2.91	2.99	4.53	6.87

* Selectivity indices (SI) for breast cancer cell lines were calculated as IC₅₀ (MCF-10A)/IC_50_ (breast cancer cells)

# Selectivity indices (SI) for other cancer cell lines were calculated as IC₅₀ (NIH/3T3)/IC_50_ (cancer cells)

#### Structure–activity relationship (SAR) analysis

Structure–activity relationship (SAR) analysis of the benzyloxymethyl-substituted imidazo[1,2-a]pyridine derivatives suggests that both electronic properties and substitution patterns of the aromatic moiety influence antiproliferative activity in a non-linear and context-dependent manner. The unsubstituted phenyl derivative **IPO1** showed relatively weak activity, indicating that aromatic substitution is generally beneficial for biological activity within this scaffold.

Among the monosubstituted derivatives, electron-donating groups such as methoxy (**IPO8**) and ethoxy (**IPO9**) were associated with strong activity, particularly against A549 and MDA-MB-231 cell lines. However, this trend is not universally consistent across all compounds, as certain electron-withdrawing derivatives also retained significant activity, indicating that electronic effects alone do not fully account for the observed biological profile. In particular, CF_3_-containing **IPO10** showed notable potency against the MDA-MB-231 cell line, highlighting a deviation from a simple electronic substituent trend and suggesting a more complex structure–activity relationship. Halogenated derivatives (**IPO3**-**6**) showed moderate activity, indicating that lipophilicity and substituent size may also contribute to activity modulation. Bulky aromatic substitution appeared to influence the antiproliferative activity within the studied series. In particular, the biphenyl derivative **IPO13** exhibited strong activity and relatively higher selectivity against A549 cells, suggesting that aromatic extension at this position may be favorable for biological activity in this scaffold. In contrast, the naphthyl derivative **IPO14** showed reduced activity and selectivity, indicating that excessive steric bulk and/or reduced conformational flexibility may adversely affect biological activity. These findings collectively suggest that steric and conformational factors may play an important role in modulating the anticancer activity of this series. However, a detailed mechanistic understanding will require further structural and computational studies.

From a design perspective, the results suggest that the most promising anticancer scaffolds are those that balance moderate lipophilicity with controlled steric demand at the aromatic substituent. While para-alkoxy derivatives (**IPO8** and **IPO9**) exhibit consistently strong activity across multiple cell lines, the notable potency of the CF_3_-substituted **IPO10** suggests that electron-withdrawing groups may be beneficial in a context-dependent manner rather than inherently unfavorable. In contrast, excessive steric bulk, as observed in the naphthyl derivative **IPO14**, appears detrimental to activity. Therefore, future optimization efforts may benefit from maintaining a compact aromatic system while fine-tuning electronic properties through moderate substituent variation rather than introducing highly bulky or rigid aromatic extensions.

#### Effects of lead compounds on BCL-2 levels, bax levels, mitochondrial membrane potential, and caspase-3 activity

Building upon the potent antiproliferative activity and favorable selectivity profiles of the lead derivatives, further mechanistic studies were conducted to elucidate their mode of action. Mitochondrial dysfunction and dysregulation of apoptotic signaling are closely associated with cancer cell death and play a central role in the execution of the intrinsic apoptotic pathway (Giampazolias and Tait 2016; Tian et al. 2024; Plati et al. 2011). Consequently, numerous small molecules have been developed to restore apoptotic signaling and overcome apoptosis resistance in cancer cells (Cui et al. 2019; Huang et al. 2018, 2019). The intrinsic apoptotic pathway is primarily regulated by the BCL-2 family of proteins, which govern mitochondrial integrity and cellular susceptibility to apoptotic stimuli (Vogler et al. 2025). In particular, the balance between anti-apoptotic BCL-2 and pro-apoptotic Bax is a critical determinant of mitochondrial outer membrane permeabilization, cytochrome c release, and subsequent caspase activation (Lin et al. 2005; Tian et al. 2024). Therefore, the effects of the selected **IPO** derivatives on key markers of the intrinsic apoptotic pathway were investigated. Specifically, BCL-2 and Bax protein levels, the Bax/BCL-2 ratio, mitochondrial membrane potential, and caspase-3 activity were evaluated to determine whether the observed cytotoxicity of the **IPO** derivatives is associated with mitochondria-mediated apoptotic cell death.

**Fig. 4:**
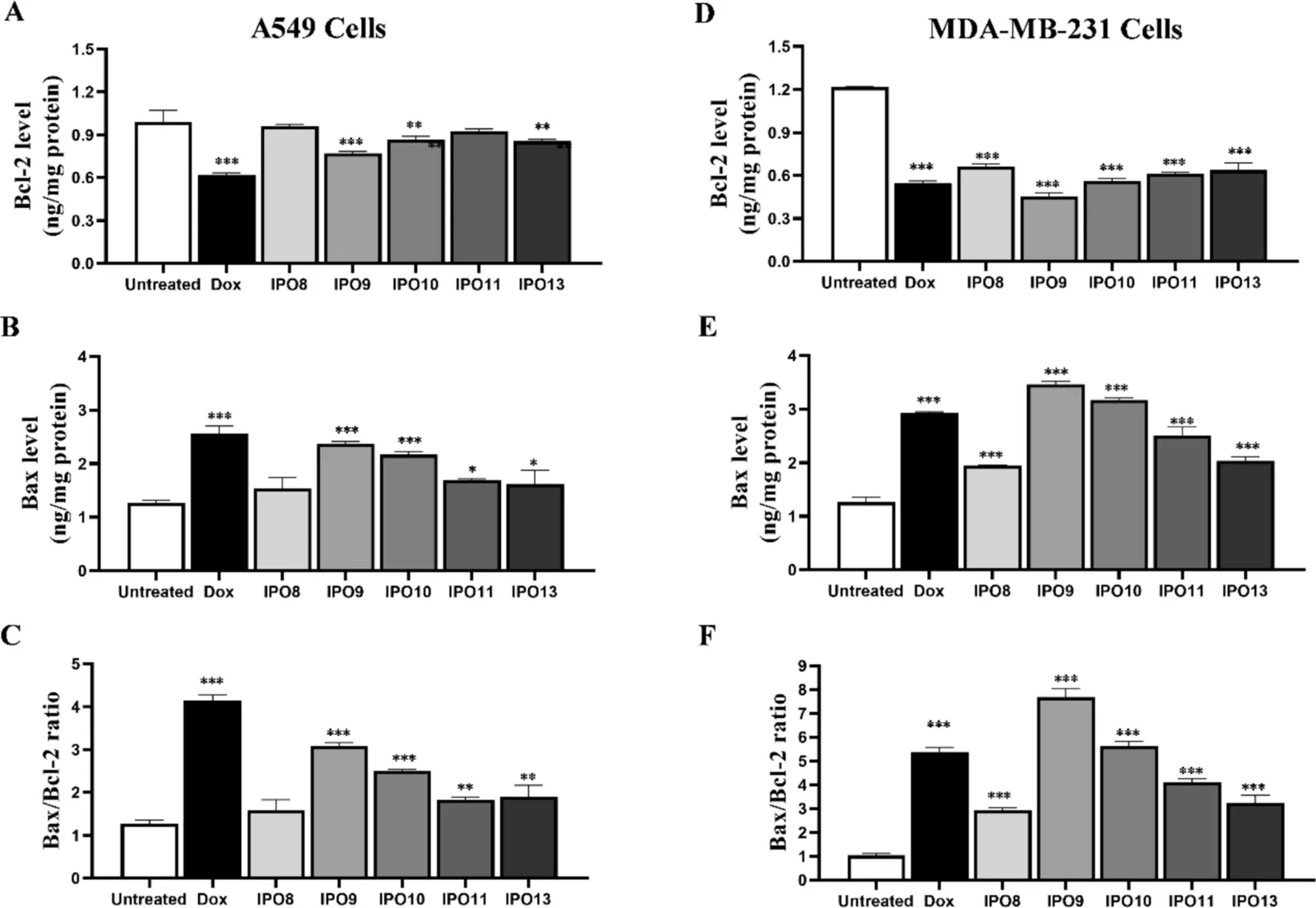
Effects of selected **IPO** derivatives on apoptosis-related markers in A549 and MDA-MB-231 cells. (**A**) BCL-2 protein levels, (**B**) Bax protein levels, (**C**) Bax/BCL-2 ratio in A549 cells following treatment with **IPO8**, **IPO9**, **IPO10**, **IPO11**, or **IPO13**. (**D**) BCL-2 protein levels, (**E**) Bax protein levels, (**F**) Bax/BCL-2 ratio in MDA-MB-231 cells following treatment with the selected **IPO** derivatives. Doxorubicin (Dox) was used as a positive control. BCL-2 and Bax protein levels were quantified by ELISA and expressed as ng/mg protein. The Bax/BCL-2 ratio was calculated from the corresponding Bax and BCL-2 values. Data are presented as mean ± SD of three independent experiments. Statistical significance was determined relative to untreated control cells (*p < 0.05, **p < 0.01, ***p < 0.0001)

Based on the cytotoxicity and selectivity results, **IPO8**, **IPO9**, **IPO10**, **IPO11**, and **IPO13** were identified as the most potent and selective derivatives and subsequently selected for mechanistic investigations. The effects of these compounds on BCL-2 and Bax protein levels in A549 and MDA-MB-231 cells were evaluated using ELISA-based assays. Because the balance between anti-apoptotic BCL-2 and pro-apoptotic Bax is a critical determinant of intrinsic apoptotic signaling, the Bax/BCL-2 ratio was also calculated. Doxorubicin was included as a positive control in all mechanistic assays. The results are presented in Fig. 4.

The ELISA-based analysis demonstrated that all tested **IPO** derivatives reduced BCL-2 protein levels, indicating their potential to induce apoptosis. These findings are consistent with previous reports demonstrating that suppression of anti-apoptotic BCL-2 signaling contributes to apoptosis induction and enhanced chemosensitivity in cancer cells (Czabotar et al. 2014; Oakes et al. 2012). In A549 cells, all compounds except **IPO8** and **IPO11** showed statistically significant inhibition compared with the untreated control. The percentage inhibition of BCL-2 expression relative to untreated cells was approximately 22.3% for **IPO9**, 12.5% for **IPO10**, and 13.6% for **IPO13**, whereas **IPO8** and **IPO11** showed only modest inhibition (2.8% and 6.6%, respectively), which was not statistically significant (Fig. 4A). As expected, doxorubicin markedly reduced BCL-2 protein levels compared with untreated cells. Among IPO derivatives, **IPO9**, which also had the lowest IC_50_ values in the cytotoxicity assays (Table 1), produced the strongest suppression of BCL-2 levels in A549 cells, suggesting that inhibition of anti-apoptotic BCL-2 signaling may contribute to its potent antiproliferative activity (Fig. 4A).

Evaluation of Bax protein levels revealed an opposite trend to that observed for BCL-2 expression. In A549 cells, doxorubicin treatment resulted in the greatest increase in Bax levels, producing an approximately 2.03-fold increase relative to untreated cells (Fig. 4B). Among the **IPO** derivatives, **IPO9** and **IPO10** exerted the strongest effects, increasing Bax expression by approximately 1.88-fold and 1.72-fold, respectively, whereas **IPO11** and **IPO13** induced more moderate increases of 1.34-fold and 1.28-fold, respectively. **IPO8** produced only a modest elevation in Bax levels (1.22-fold) compared with untreated cells (Fig. 4B). Notably, **IPO9**, which also showed the most pronounced suppression of BCL-2 expression among the **IPO** derivatives, induced the strongest Bax expression, further supporting its pro-apoptotic activity. Consequently, all tested derivatives increased the Bax/BCL-2 ratio relative to untreated cells (Fig. 4C). Among the **IPO** derivatives, **IPO9** exhibited the strongest response, increasing the Bax/BCL-2 ratio by approximately 2.41-fold, followed by **IPO10** (1.97-fold). More moderate increases were observed for **IPO13** (1.49-fold), **IPO11** (1.43-fold), and **IPO8** (1.25-fold). Notably, the pronounced increase in the Bax/BCL-2 ratio induced by **IPO9** was consistent with its strong suppression of BCL-2 expression and marked induction of Bax levels, further supporting its pro-apoptotic activity in A549 cells.

In MDA-MB-231 cells, all tested compounds significantly reduced BCL-2 expression compared with the untreated group (Fig. 4D). The percentage inhibition values were approximately 45.5% (**IPO8**), 62.9% (**IPO9**), 54.0% (**IPO10**), 49.6% (**IPO11**), and 47.6% (**IPO13**), indicating pronounced inhibition across all derivatives. Doxorubicin, used as a positive control, reduced BCL-2 levels by approximately 55.3% relative to untreated cells (Fig. 4D). Notably, **IPO9** produced the strongest suppression of BCL-2 expression among the **IPO** derivatives. The reduction in BCL-2 levels induced by **IPO9** was significantly greater than that observed with doxorubicin (p < 0.01), suggesting a particularly strong modulatory effect of **IPO9** on anti-apoptotic BCL-2 signaling. In addition, **IPO10** reduced BCL-2 expression to levels comparable to doxorubicin, further supporting the potent BCL-2-modulating properties of the lead compounds. Importantly, although **IPO8** did not show statistically significant inhibition in A549 cells, it exhibited a highly significant inhibitory effect in MDA-MB-231 cells (45.5%), highlighting a pronounced cell line-dependent response (Fig. 4D). Notably, BCL-2 suppression was markedly more pronounced in MDA-MB-231 cells than in A549 cells, further supporting the enhanced sensitivity of triple-negative breast cancer cells to the **IPO** derivatives. This observation may be therapeutically relevant given the aggressive phenotype and limited targeted treatment options associated with triple-negative breast cancer (Raman et al. 2025).

Consistent with the reduction in BCL-2 levels, all tested **IPO** derivatives significantly increased Bax expression in MDA-MB-231 cells (Fig. 4E). Doxorubicin treatment increased Bax expression by approximately 2.31-fold relative to untreated cells, whereas **IPO9** and **IPO10** produced even greater increases of 2.73-fold and 2.49-fold, respectively. Among the **IPO** derivatives, **IPO9** exhibited the strongest Bax induction and generated significantly higher Bax levels than the positive control, doxorubicin (p < 0.01). Notably, **IPO10** also produced significantly greater Bax expression than doxorubicin (p < 0.05), highlighting the pronounced pro-apoptotic activity of these lead compounds in MDA-MB-231 cells.

As a result, all compounds markedly increased the Bax/BCL-2 ratio relative to untreated cells, with **IPO9** again showing the highest ratio among the tested derivatives (Fig. 4F). The mean Bax/BCL-2 ratio increased from 1.04 in untreated cells to 7.68 after **IPO9** treatment, whereas doxorubicin increased it to 5.39. **IPO10** also markedly increased the Bax/BCL-2 ratio (5.62), yielding values comparable to those observed with doxorubicin. Notably, **IPO9** produced the highest Bax/BCL-2 ratio among all treatment groups, exceeding that observed with the positive control doxorubicin and further supporting its pronounced pro-apoptotic activity in MDA-MB-231 cells. In addition, the increase in Bax/BCL-2 ratio was substantially greater in MDA-MB-231 cells than in A549 cells, suggesting that triple-negative breast cancer cells are more susceptible to apoptosis induction by these compounds.

Collectively, these findings suggest that the **IPO** derivatives may function as modulators of BCL-2-mediated survival signaling, particularly in triple-negative breast cancer cells, which exhibited greater sensitivity to BCL-2 suppression. Moreover, the concurrent decrease in BCL-2 levels, increase in Bax expression, and elevation of the Bax/BCL-2 ratio support activation of mitochondria-mediated intrinsic apoptosis. Since the balance between anti-apoptotic BCL-2 and pro-apoptotic Bax is a critical determinant of mitochondrial outer membrane integrity and apoptotic commitment (Martinou and Youle 2011), the observed shift toward a higher Bax/BCL-2 ratio suggests enhanced susceptibility of cancer cells to apoptosis. Notably, **IPO9** exerted stronger effects on both BCL-2 suppression and Bax induction than the positive control doxorubicin in MDA-MB-231 cells, highlighting its pronounced impact on apoptosis-related signaling pathways. Taken together, these findings indicate that modulation of BCL-2 family proteins may contribute to the apoptotic effects induced by the lead **IPO** derivatives. Since changes in the BCL-2/Bax balance are closely linked to mitochondrial dysfunction and downstream caspase activation (Vogler et al. 2025), evaluating the organelle-level impact became essential. Therefore, in the next stage of our mechanistic investigation, the effects of the selected **IPO** derivatives on mitochondrial membrane potential were further investigated.

**Fig. 5:**
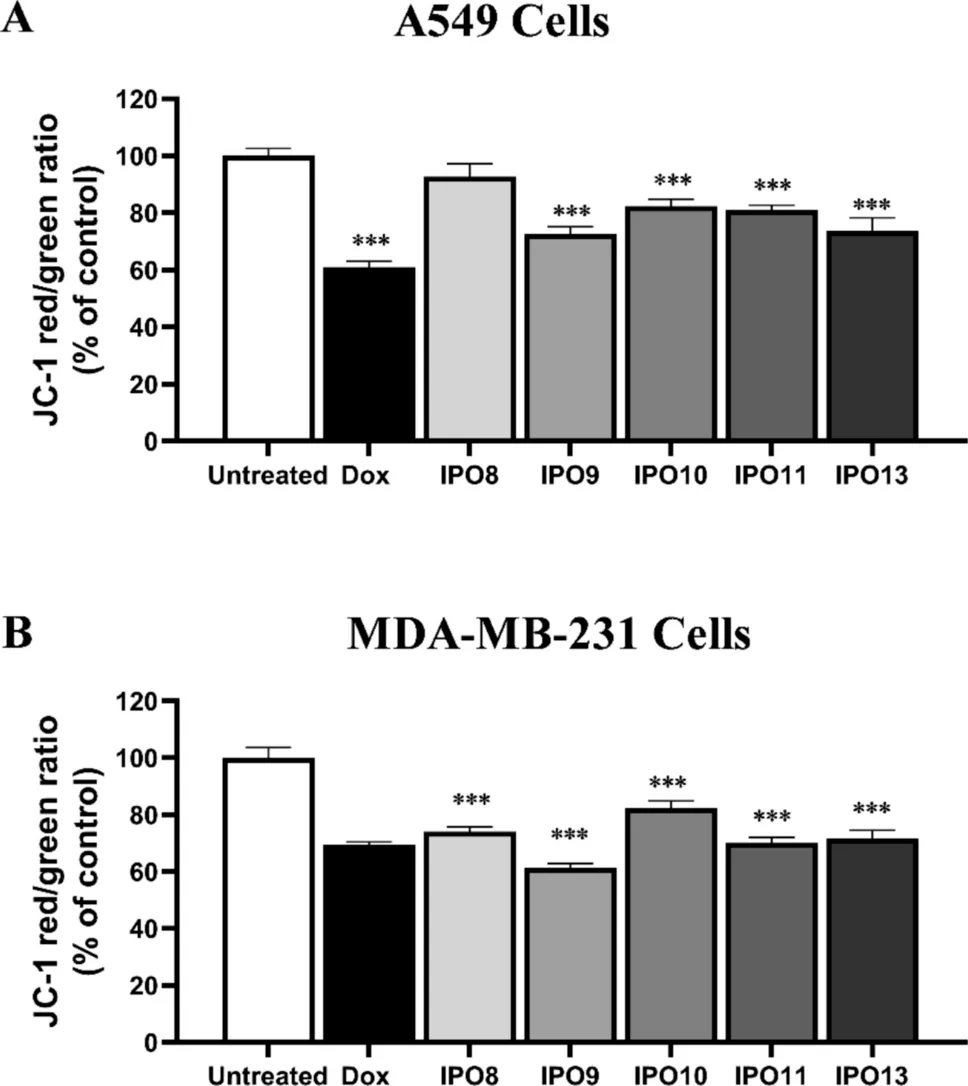
Effects of selected **IPO** derivatives on mitochondrial membrane potential in (**A**) A549 and (**B**) MDA-MB-231 cells. Cells were treated with **IPO8**, **IPO9**, **IPO10**, **IPO11**, or **IPO13**. Doxorubicin (Dox) was included as a positive control. Mitochondrial membrane potential was assessed using a JC-1 fluorescent assay and expressed as the JC-1 red/green fluorescence ratio (% of untreated control). A decrease in the JC-1 red/green fluorescence ratio indicates mitochondrial membrane depolarization. Data are presented as mean ± SD of three independent experiments. Statistical significance was determined relative to untreated control cells (***p < 0.0001)

To further investigate whether the observed reduction in BCL-2 levels was associated with mitochondrial dysfunction, the effects of the selected **IPO** derivatives on mitochondrial membrane potential were assessed using a JC-1-based fluorescent assay. The results are shown in Fig. 5.

Treatment with the selected **IPO** derivatives or doxorubicin reduced the JC-1 red-to-green fluorescence ratio in both A549 and MDA-MB-231 cells, indicating loss of mitochondrial membrane potential and mitochondrial depolarization (Fig. 5). In A549 cells, doxorubicin produced the greatest reduction in the JC-1 fluorescence ratio, decreasing it to approximately 61% of the control value (Fig. 5A). Among the **IPO** derivatives, **IPO9** and **IPO13** exerted the most pronounced effects, reducing the JC-1 fluorescence ratio to approximately 73% and 74% of the control level, respectively. **IPO10** and **IPO11** also significantly decreased mitochondrial membrane potential, whereas **IPO8** showed only a mild, statistically nonsignificant effect (Fig. 5A). These findings indicate that the lead **IPO** derivatives promote mitochondrial depolarization, consistent with their effects on BCL-2 suppression, Bax induction, and Bax/BCL-2 ratio modulation.

In MDA-MB-231 cells, all tested compounds reduced mitochondrial membrane potential, with **IPO9** again producing the most pronounced effect, reducing the JC-1 red/green fluorescence ratio to approximately 61% of the control level (Fig. 5B). Doxorubicin reduced the ratio to approximately 69% of control, whereas **IPO8**, **IPO11**, and **IPO13** produced intermediate effects, reducing the ratio to approximately 74%, 70%, and 72% of control, respectively. **IPO10** induced a more moderate decrease in mitochondrial membrane potential, reducing the JC-1 ratio to approximately 82% of control. Notably, the mitochondrial depolarization induced by **IPO9** was significantly greater than that observed with doxorubicin (*p* < 0.05), further supporting its pronounced effect on mitochondria-mediated apoptotic signaling (Fig. 5B). Similar to the BCL-2, Bax, and Bax/BCL-2 ratio results, mitochondrial depolarization was generally more pronounced in MDA-MB-231 cells than in A549 cells (Figs. 4 and 5), further supporting the greater sensitivity of triple-negative breast cancer cells to the **IPO** derivatives.

Loss of mitochondrial membrane potential is widely recognized as a key event in the intrinsic apoptotic pathway and reflects disruption of mitochondrial function (Chipuk et al. 2006). During apoptosis, mitochondrial outer membrane permeabilization promotes the release of pro-apoptotic factors, including cytochrome c, leading to apoptosome formation and subsequent activation of the caspase cascade. Consequently, mitochondrial depolarization is closely associated with caspase activation and progression toward apoptotic cell death (Chipuk et al. 2006; Wang and Youle 2009; Huang et al. 2019). Therefore, the observed disruption of mitochondrial membrane potential is consistent with the involvement of mitochondria-mediated apoptotic mechanisms in the cytotoxic effects of the **IPO** derivatives. Notably, in MDA-MB-231 cells, **IPO9** produced the most pronounced effects on BCL-2 suppression, Bax induction, Bax/BCL-2 ratio elevation, and mitochondrial membrane depolarization among the tested **IPO** derivatives, while also exhibiting stronger effects than doxorubicin on several apoptosis-related parameters (Figs. 4 and 5).

**Fig. 6:**
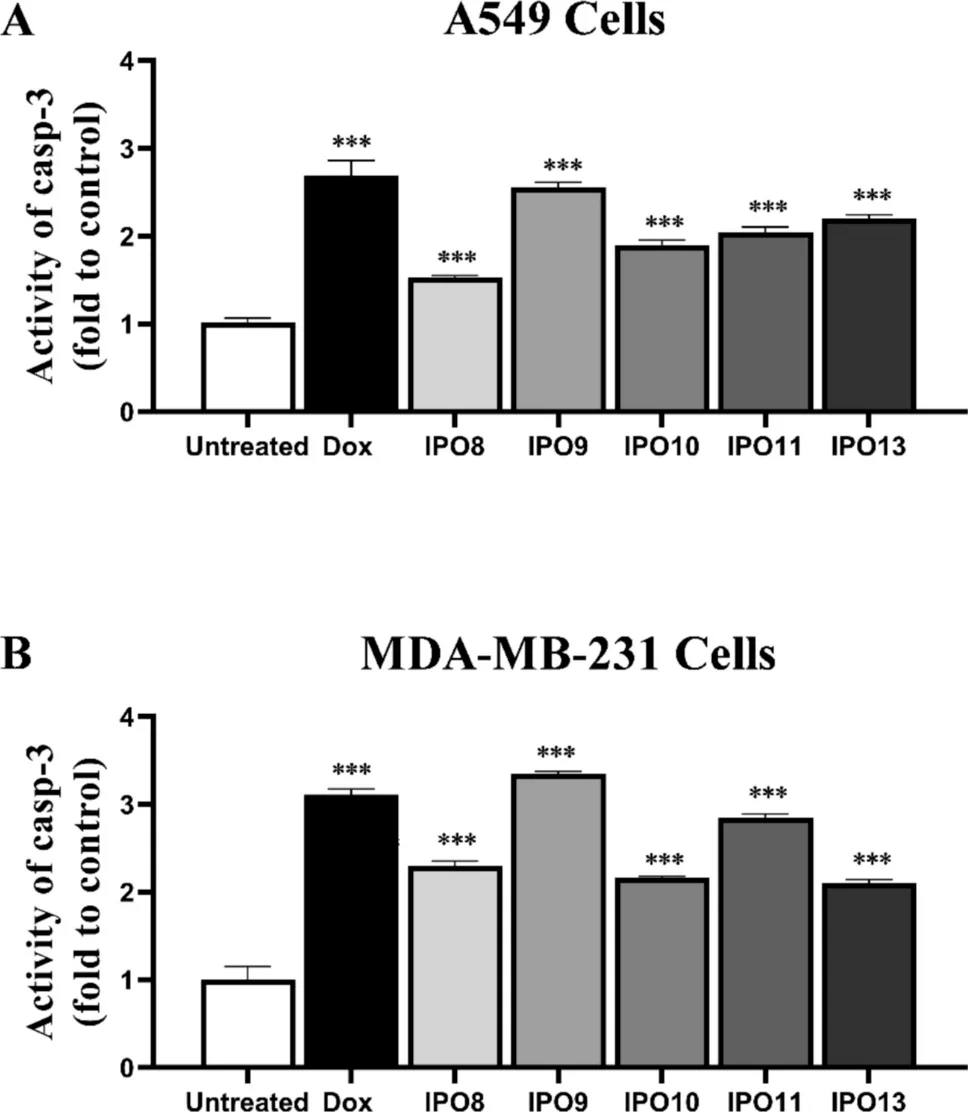
Effects of selected **IPO** derivatives on caspase-3 activity in (**A**) A549 and (**B**) MDA-MB-231 cells. Cells were treated with **IPO8**, **IPO9**, **IPO10**, **IPO11**, or **IPO13**. Doxorubicin (Dox) was included as a positive control. Caspase-3 activity was determined using a colorimetric assay and expressed as fold change relative to untreated control cells. Increased caspase-3 activity indicates activation of apoptotic signaling. Data are presented as mean ± SD of three independent experiments. Statistical significance was determined relative to untreated control cells (***p < 0.0001)

Caspase-3 activity assays further supported the pro-apoptotic effects of the selected **IPO** derivatives. Activation of caspase-3 is widely recognized as a critical downstream event in the execution phase of apoptosis (Elmore 2007). In A549 cells, all tested compounds significantly increased caspase-3 activity compared with the untreated control (Fig. 6A). Doxorubicin, used as a positive control, increased caspase-3 activity to approximately 2.69-fold of the control level (Fig. 6A). Caspase-3 activity reached approximately 1.53-fold, 2.55-fold, 1.90-fold, 2.05-fold, and 2.20-fold of control following treatment with **IPO8**, **IPO9**, **IPO10**, **IPO11**, and **IPO13**, respectively (Fig. 6A). Among the **IPO** derivatives, **IPO9** produced the strongest activation of caspase-3, approaching the effect observed with doxorubicin. This finding is consistent with its potent cytotoxic activity, marked suppression of BCL-2, induction of Bax expression, elevation of the Bax/BCL-2 ratio, and pronounced mitochondrial membrane depolarization (Table 1, Figs. 4 and 5).

In MDA-MB-231 cells, a more robust induction of caspase-3 activity was observed across all compounds. Doxorubicin increased caspase-3 activity to approximately 3.11-fold of the control level (Fig. 6B). Caspase-3 activity reached approximately 2.30-fold, 3.35-fold, 2.16-fold, 2.85-fold, and 2.10-fold of control following treatment with **IPO8, IPO9**, **IPO10**, **IPO11**, and **IPO13**, respectively. Notably, **IPO9** again produced the highest level of caspase-3 activation among the tested **IPO** derivatives and induced significantly greater caspase-3 activity than doxorubicin (p < 0.05), further supporting its pronounced pro-apoptotic activity in MDA-MB-231 cells. The observed increase in caspase-3 activity following mitochondrial membrane depolarization further supports the involvement of mitochondria-mediated intrinsic apoptotic signaling.

Taken together, the coordinated suppression of BCL-2, induction of Bax expression, elevation of the Bax/BCL-2 ratio, mitochondrial membrane depolarization, and subsequent caspase-3 activation indicate a mechanistically coherent apoptotic response induced by the **IPO** derivatives (Figs. 4, 5, 6). Among the tested derivatives, **IPO9** consistently exhibited the most pronounced pro-apoptotic profile, particularly in MDA-MB-231 cells, where it produced the strongest modulation of apoptosis-related parameters and, in several assays, demonstrated greater effects than the positive control doxorubicin.

### Molecular docking studies

To further elucidate the molecular basis of the observed biological activity and apoptosis induction, molecular docking studies were performed against BCL-2 (PDB ID: 4MAN). In light of the significant downregulation of BCL-2 *in vitro*, the interactions of the selected compounds (**IPO8**, **IPO9**, **IPO10**, **IPO11**, and **IPO13**) were systematically investigated at the molecular level to evaluate their binding modes and interaction profiles within the BCL-2 binding pocket. Docking analysis was conducted to provide structural insight into the observed biological activity (Table 3).

**Table 3:** Docking scores of synthesized compounds **IPO8, IPO9, IPO10, IPO11, IPO13, and co-ligand** against BCL-2 calculated using AutoDock 4.2

Compounds	BCL-2 (PDB ID: 4MAN)		
	Binding affinity (kcal/mol)	Cluster RMSD (Å)	Interacted amino acid residues
IPO8	6.649	0.53	Phe101, Tyr105, Met112, Val130, Leu134, Ala146, Val153
IPO9	6.828	0.14	Phe101, Tyr105, Phe109, Met112, Val130, Leu134, Ala146, Val153
IPO10	7.015	0.14	Phe101, Tyr105, Asp108, Phe109, Met112, Val130, Leu134, Ala146, Val153
IPO11	6.558	0.59	Phe101, Tyr105, Met112, Val130, Leu134, Ala146, Val153
IPO13	7.140	0.51	Tyr105, Met112, Leu134, Ala146, Val153
Navitoclax	8.070	0.86	Ala97, Asp100, Phe101, Tyr105, Met112, Leu134, Val145, Tyr199

**Fig. 7:**
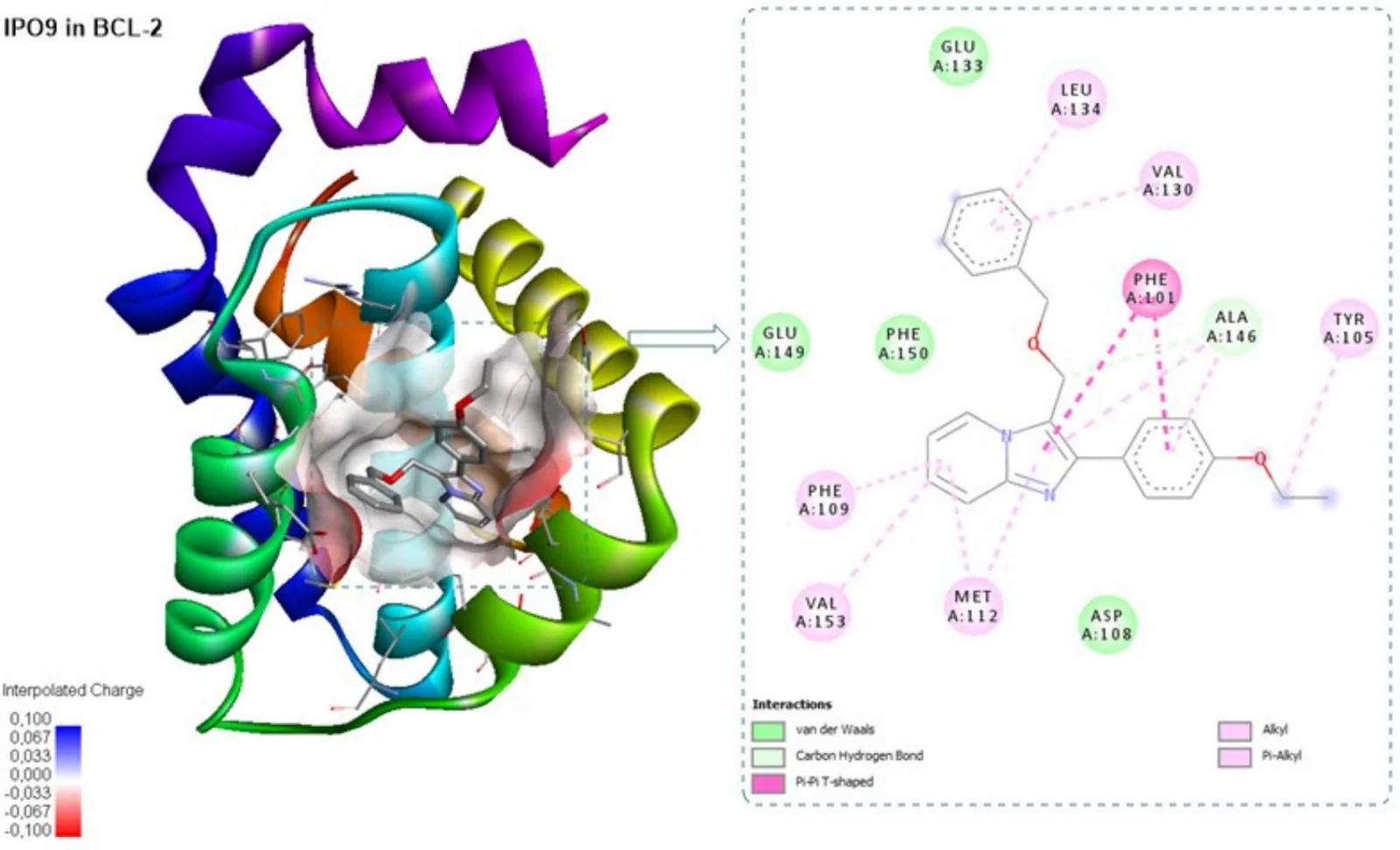
3D and 2D interaction profiles of **IPO9** in the BCL-2 active site (PDB ID: 4MAN)

Docking results against BCL-2 revealed binding affinities ranging from 6.558 to 7.140 kcal/mol. **IPO13** exhibited the highest binding affinity (7.140 kcal/mol; RMSD: 0.51 Å), followed by **IPO10** (7.015 kcal/mol; RMSD: 0.14 Å). **IPO9** showed a binding affinity of 6.828 kcal/mol, outperforming **IPO8** (6.649 kcal/mol; RMSD: 0.53 Å) and **IPO11** (6.558 kcal/mol; RMSD: 0.59 Å). Notably, **IPO9** displayed one of the lowest RMSD values (0.14 Å), indicating a highly stable and reproducible binding conformation. In comparison, the reference ligand Navitoclax exhibited a higher binding energy (8.070 kcal/mol) but a higher RMSD (0.86 Å), suggesting less consistent pose convergence under the same conditions. As depicted in Fig. 7, **IPO9** is effectively accommodated within the hydrophobic groove of BCL-2 and forms key stabilizing interactions, including hydrophobic contacts. Importantly, the interaction pattern of **IPO9** overlaps with that of the co-crystallized ligand, indicating that it can mimic native ligand interactions within the binding pocket.

Overall, the docking analysis indicates that the selected derivatives interact with BCL-2 in a manner comparable to that of the co-crystallized ligand. Among them, **IPO9** stands out due to its favorable binding affinity and consistently low RMSD values, suggesting a stable and reproducible binding mode within the BCL-2 binding pocket. Together with its superior *in vitro* activity, these findings support the potential involvement of **IPO9** in BCL-2-related apoptotic pathways and highlight it as a promising scaffold for further investigation.

#### Pre-ADMET analysis of the selected compounds

The pre-ADMET evaluation of the selected *in vitro* active compounds (**IPO8, IPO9, IPO10, IPO11,** and **IPO13**) was conducted to gain early insight into their pharmacokinetic behavior, drug-likeness, and safety-related profiles. Overall, all selected compounds satisfied Lipinski’s rule of five, indicating favorable oral drug-like properties and supporting their potential for further development (Table 4).

**Table 4:** The drug-likeness and predicted ADMET results for the compounds **IPO8, IPO9, IPO10, IPO11,** and **IPO13**

Entry	Some important ADMET Parameters					
	Lipinski Rules	Caco-2 permeability, 10^–6^ cm/s	VDss, L/kg	CYP substrate or inhibitor*	CL_plasma_ ml/min/kg	AMES Toxicity
IPO8	Accepted	−4.824	1.826	CYP1A2, CYP2C19, CYP2C9	6.323	0.57
IPO9	Accepted	−4.771	1.983	CYP1A2, CYP2C19, CYP2C9	6.109	0.578
IPO10	Accepted	−4.914	4.667	CYP1A2, CYP2C19, CYP2C9	7.01	0.21
IPO11	Accepted	−4.914	3.321	CYP1A2, CYP2C19, CYP2C9	5.273	0.19
IPO13	Accepted	−4.869	2.524	CYP1A2, CYP2C19	4.949	0.653

The predicted Caco-2 permeability values (− 4.914 to − 4.771) indicate comparable, moderate intestinal permeability across the series. Among the compounds, **IPO9** showed the highest predicted permeability (− 4.771), indicating relatively improved intestinal transport within this dataset, although the overall differences among compounds are limited. The predicted volume of distribution (VDss) values ranged from 1.826 to 4.667 L/kg, reflecting variability in tissue distribution profiles. **IPO9** displayed a VDss value of 1.983 L/kg, suggesting a moderate distribution pattern relative to the other derivatives. From a metabolic perspective, all compounds were predicted to interact with one or more major cytochrome P450 isoforms (CYP1A2, CYP2C19, and CYP2C9). These interactions are commonly observed for small-molecule drug-like scaffolds and should be interpreted as potential metabolic liabilities that require experimental confirmation rather than definitive metabolic behavior. Plasma clearance values were within a moderate range (4.949–7.01 mL/min/kg), indicating no extreme predictions of rapid elimination across the series. The AMES toxicity prediction values ranged from 0.19 to 0.653, suggesting low-to-moderate mutagenic risk across the dataset. **IPO9** showed a value of 0.578, comparable to **IPO8** but higher than **IPO10** and **IPO11**. Overall, the evaluated compounds exhibit generally acceptable in silico ADMET profiles consistent with drug-like chemical space. **IPO9** demonstrates relatively balanced predicted physicochemical and pharmacokinetic properties within the series; however, these findings should not be interpreted as evidence of optimized drug candidacy but rather as preliminary guidance for further lead optimization and experimental evaluation.

## Conclusion

This study describes the successful design, synthesis, and biological evaluation of a novel series of benzyloxymethyl-substituted imidazo[1,2-a]pyridine derivatives (**IPO1-14**) as potential anticancer agents. The developed synthetic strategy enabled efficient preparation of structurally diverse analogs and facilitated systematic structure–activity relationship analysis. Biological evaluation demonstrated that anticancer activity was strongly influenced by aromatic substitution patterns, with para-alkoxy-substituted derivatives exhibiting the most potent antiproliferative activity and selectivity. Among the synthesized compounds, **IPO9** emerged as the most active derivative, displaying pronounced cytotoxicity and favorable selectivity, particularly against A549 non-small cell lung cancer and MDA-MB-231 triple-negative breast cancer cells. Mechanistic studies revealed that the lead derivatives induced mitochondria-mediated intrinsic apoptosis through suppression of BCL-2, induction of Bax expression, elevation of the Bax/BCL-2 ratio, disruption of mitochondrial membrane potential, and activation of caspase-3. Notably, **IPO9** consistently produced the strongest effects across cytotoxicity and apoptosis-related assays. In MDA-MB-231 cells, **IPO9** exhibited stronger modulation of several apoptosis-related parameters than doxorubicin under the experimental conditions employed. Molecular docking studies further demonstrated favorable interactions between **IPO9** and the key apoptotic target BCL-2, while in silico ADMET analyses suggested promising drug-like and pharmacokinetic properties. Overall, these findings identify **IPO9** as a promising lead candidate for further optimization and provide a valuable framework for developing next-generation imidazo[1,2-a]pyridine-based anticancer agents. Further in vivo and translational investigations are warranted to validate its therapeutic potential. As a limitation of the present study, the lead **IPO** derivatives exhibited higher IC_50_ values than the reference drug doxorubicin across the evaluated cancer cell lines. Nevertheless, mechanistic studies demonstrated significant modulation of apoptosis-related pathways, particularly by **IPO9**. Therefore, future optimization of the **IPO** scaffold should focus on improving antiproliferative potency while preserving its favorable apoptosis-inducing activity and selectivity profile.

## Experimental section

### Chemistry

#### General information

^1^H and ^13^C NMR spectra were recorded on a Varian-Agilent Inova instrument (400 and 100 MHz, respectively) using Me_4_Si (TMS) as the internal standard. Melting points were determined on a Stuart Melting Point (SMP30) analyzer using open glass capillaries. Column chromatography was performed on silica gel (60 mesh, Silycycle). Commercially available materials were used without further purification. Mass spectra were recorded using an Agilent 6230 LC/TOF spectrometer with a dual AJS ESI method. Analysis results have a maximum deviation of ± 0.4 from the calculated theoretical values.

#### General procedure for the synthesis of α-bromoketones (B(1–14))

Copper(II) bromide (1.2 mmol) was added to a solution of aryl methyl ketone derivatives **A(1–14)** (5 mmol) in ethyl acetate-chloroform (1:1, 30 mL). The reaction mixture was refluxed for 6 h and then stirred at room temperature overnight. The progress of the reaction was monitored by TLC. Upon completion, the reaction mixture was filtered and the filtrate was concentrated under reduced pressure to afford the corresponding α-bromoketones **B(1–14**) in 90–95% yield, which were used in the next step without further purification (Kuzu and Demir 2025).

#### General procedure for the synthesis of imidazo[1,2-a]pyridine derivatives (C(1–14))

A mixture of α-bromoketones **B(1–14)** (1 mmol), 2-aminopyridine (1 mmol), and sodium bicarbonate (1.5 mmol) in ethanol (10 mL) was stirred at 65 °C for 4 h and then at room temperature for an additional 2 h. The reaction progress was monitored by TLC. After completion, the solvent was evaporated under reduced pressure. Water (20 mL) was added to the residue and the mixture was stirred at room temperature for 30 min, resulting in precipitation of the product. The solid was collected by filtration and dried, affording the corresponding imidazo[1,2-a]pyridine derivatives C(1–14) (Kuzu and Demir 2025).

#### General procedure for the synthesis of formyl derivatives (D(1–14))

A precooled solution of DMF (3 mmol) and POCl_3_ (3 mmol) was prepared by dropwise addition at 0 °C and stirred for 15 min. A solution of imidazo[1,2-a]pyridine derivatives **C(1–14)** (1 mmol) in DMF (3 mL) was added dropwise to the reaction mixture, and the resulting mixture was heated at 80 °C for 3 h. After completion, the reaction mixture was cooled to room temperature and poured into an ice-water mixture (20 mL). The mixture was stirred for 30 min, then neutralized to pH 8 using 10% aqueous NaOH. The precipitated solid was filtered, washed with water, and dried to afford the corresponding aldehyde derivatives **D(1–14)** (Kuzu et al. 2026).

#### General procedure for the synthesis of alcohol derivatives (E(1–14))

To a solution of aldehyde derivatives **D(1–14)** (1 mmol) in ethanol (10 mL), sodium borohydride (1.5 mmol) was added portionwise at room temperature. The reaction mixture was stirred at 25 °C for 2 h, and the progress was monitored by TLC. Upon completion, the reaction was quenched with water (10 mL). The solvent was removed under reduced pressure, and the residue was extracted with ethyl acetate (3 × 15 mL). The combined organic layers were dried over anhydrous sodium sulfate and concentrated to give the corresponding alcohol derivatives **E(1–14)**, which were used in the next step without further purification (Kuzu et al. 2023).

#### General procedure for the synthesis of final compounds ((1–14))

To a stirred solution of alcohol derivatives **E(1–14)** (1 mmol) in dry DMF (10 mL), sodium hydride (60% dispersion in mineral oil, 1.2 mmol) was added portionwise at 0 °C under inert atmosphere. The mixture was stirred at the same temperature for 30 min, followed by the dropwise addition of the appropriate benzyl bromide derivative (1.2 mmol). The reaction mixture was then allowed to warm to room temperature and stirred for 4 h. The progress of the reaction was monitored by TLC. After completion, the reaction was quenched carefully with cold water (10 mL), and the mixture was extracted with ethyl acetate (3 × 15 mL). The combined organic layers were dried over anhydrous sodium sulfate and concentrated under reduced pressure. The crude product was purified by column chromatography on silica gel (n-hexane/EtOAc) to afford the target compounds (**IPO1**-**14**) (Deniz et al. 2025).

##### 3-((benzyloxy)methyl)−2-phenylimidazo[1,2-a]pyridine (IPO1)

White solid, M.p.: 106–108 °C, Yield: 89%, Rf: 0.125 (ethyl acetate/n-hexane, 1/3). ^1^H NMR (400 MHz, CDCl_3_) δ 8.09 (d, J = 6.8 Hz, 1H, Ar–H), 7.73 (d, J = 7.5 Hz, 2H, Ar–H), 7.64 (d, J = 7.9 Hz, 1H, Ar–H), 7.47–7.26 (m, 8H, Ar–H), 7.21 (t, J = 7.9 Hz, 1H, Ar–H), 6.81 (t, J = 6.8 Hz, 1H, Ar–H), 4.90 (s, 2H, -OCH_2_-), 4.56 (s, 2H, -OCH_2_-). ^13^C NMR (100 MHz, CDCl_3_) δ 145.8, 145.3, 137.4, 134.1, 128.7, 128.6, 128.5, 128.2, 128.0, 127.9, 125.1, 124.3, 117.5, 116.7, 112.4, 72.0, 60.8. LC-TOF/MS [M + H]^+^; Calculated for C_21_H_19_N_2_O: 315.1492, Found: 315.1698.

##### 3-((benzyloxy)methyl)−2-(4-fluorophenyl)imidazo[1,2-a]pyridine (IPO2)

White solid, M.p.: 119–121 °C, Yield: 80%, Rf: 0.15 (ethyl acetate/n-hexane, 1/3). ^1^H NMR (400 MHz, CDCl_3_) δ 8.09 (d, J = 6.8 Hz, 1H, Ar–H), 7.76–7.56 (m, 3H, Ar–H), 7.39–7.19 (m, 6H, Ar–H), 7.09 (t, J = 8.6 Hz, 2H, Ar–H), 6.83 (t, J = 6.8 Hz, 1H, Ar–H), 4.86 (s, 2H, -OCH_2_-), 4.57 (s, 2H, -OCH_2_-). ^13^C NMR (100 MHz, CDCl_3_) δ 162.7 (d, J = 247.3 Hz), 145.2, 144.8, 137.3, 130.3 (d, J = 8.2 Hz), 130.1 (d, J = 3.2 Hz), 128.6, 128.3, 128.1, 125.3, 124.2, 117.4, 116.5, 115.5 (d, J = 21.5 Hz), 112.6, 72.1, 60.6. LC-TOF/MS [M + H]^+^; Calculated for C_21_H_18_FN_2_O: 333.1398, Found: 333.1552.

##### 3-((benzyloxy)methyl)−2-(3,4-difluorophenyl)imidazo[1,2-a]pyridine (IPO3)

Red viscous liquid Yield: 91%, Rf: 0.25 (ethyl acetate/n-hexane,1/3). ^1^H NMR (400 MHz, CDCl_3_) δ 8.13 (d, J = 6.7 Hz, 1H, Ar–H), 7.69–7.62 (m, 2H, Ar–H), 7.54–7.15 (m, 8H, Ar–H), 6.91 (t, J = 6.7 Hz, 1H, Ar–H), 4.91 (s, 2H, -OCH_2_-), 4.65 (s, 2H, -OCH_2_-). ^13^C NMR (100 MHz, CDCl_3_) δ 151.5, 149.1, 145.2, 143.6, 137.1, 131.0, 128.6, 128.2, 125.6, 124.6 (dd, J = 3.5 Hz, J = 6.3 Hz), 124.2, 117.5, 117.4 (dd, J = 3.5 Hz, J = 6.3 Hz), 116.8, 112.8, 72.2, 60.4. LC-TOF/MS [M + H]^+^; Calculated for C_21_H_17_F_2_N_2_O: 351.1303, Found: 351.1502.

##### 3-((benzyloxy)methyl)−2-(4-chlorophenyl)imidazo[1,2-a]pyridine (IPO4)

Gray solid, M.p.: 127–128 °C, Yield: 88%, Rf: 0.30 (ethyl acetate/n-hexane, 1/3). ^1^H NMR (400 MHz, CDCl_3_) δ 8.09 (d, J = 6.8 Hz, 1H, Ar–H), 7.72–7.56 (m, 3H, Ar–H), 7.40–7.19 (m, 8H, Ar–H), 6.84 (t, J = 6.8 Hz, 1H, Ar–H), 4.87 (s, 2H, -OCH_2_-), 4.57 (s, 2H, -OCH_2_-). ^13^C NMR (100 MHz, CDCl_3_) δ 145.3, 144.6, 137.3, 133.9, 132.5, 129.8, 128.8, 128.6, 128.2, 128.1, 125.3, 124.2, 117.5, 116.8, 112.6, 72.1, 60.6. LC-TOF/MS [M + H]^+^; Calculated for C_21_H_18_ClN_2_O: 349.1102, Found: 349.1287.

##### 3-((benzyloxy)methyl)−2-(3,4-dichlorophenyl)imidazo[1,2-a]pyridine (IPO5)

White solid, M.p.: 111–112 °C, Yield: 85%, Rf: 0.25 (ethyl acetate/n-hexane, 1/3). ^1^H NMR (400 MHz, CDCl_3_) δ 8.08 (d, J = 6.8 Hz, 1H, Ar–H), 7.90 (s, 1H, Ar–H), 7.64 (d, J = 9.0 Hz, 1H, Ar–H), 7.55–7.53 (m, 1H, Ar–H), 7.45 (d, J = 8.3 Hz, 1H, Ar–H), 7.41–7.20 (m, 6H, Ar–H), 6.86 (t, J = 6.8 Hz, 1H, Ar–H), 4.86 (s, 2H, -OCH_2_-), 4.60 (s, 2H, -OCH_2_-). ^13^C NMR (100 MHz, CDCl_3_) δ 145.2, 143.2, 137.1, 134.0, 132.8, 132.1, 130.5, 130.3, 128.6, 128.3, 128.2, 127.6, 125.7, 124.2, 117.5, 117.2, 112.9, 72.3, 60.4. LC-TOF/MS [M + H]^+^; Calculated for C_21_H_17_Cl_2_N_2_O: 383.0712, Found: 383.0924.

##### 3-((benzyloxy)methyl)−2-(4-bromophenyl)imidazo[1,2-a]pyridine (IPO6)

Brown solid, M.p.: 115–117 °C, Yield: 89%, Rf: 0.225 (ethyl acetate/n-hexane, 1/3). ^1^H NMR (400 MHz, CDCl_3_) δ 8.08 (d, J = 6.2 Hz, 1H, Ar–H), 7.66–7.45 (m, 5H, Ar–H), 7.38–7.19 (m, 6H, Ar–H), 6.83 (t, J = 6.2 Hz, 1H, Ar–H), 4.86 (s, 2H, -OCH_2_-), 4.56 (s, 2H, -OCH_2_-). ^13^C NMR (100 MHz, CDCl_3_) δ 145.3, 144.6, 137.2, 133.0, 131.7, 130.1, 128.6, 128.2, 128.1, 125.4, 124.2, 122.2, 117.5, 116.8, 112.6, 72.1, 60.5. LC-TOF/MS [M + H]^+^; Calculated for C_21_H_18_BrN_2_O: 393.0597, Found: 393.0761.

##### 3-((benzyloxy)methyl)−2-(p-tolyl)imidazo[1,2-a]pyridine (IPO7)

Gray solid, M.p.: 107–109 °C, Yield: 90%, Rf: 0.20 (ethyl acetate/n-hexane, 1/3). ^1^H NMR (400 MHz, CDCl_3_) δ 8.09 (d, J = 6.8 Hz, 1H, Ar–H), 7.65–7.61 (m, 3H, Ar–H), 7.35–7.26 (m, 5H, Ar–H), 7.24–7.22 (m, 3H, Ar–H), 6.81 (t, J = 6.8 Hz, 1H, Ar–H), 4.90 (s, 2H, -OCH_2_-), 4.56 (s, 2H, -OCH_2_-), 2.39 (s, 3H, Ar-CH_3_). ^13^C NMR (100 MHz, CDCl_3_) δ 145.8, 145.2, 137.8, 137.5, 131.1, 129.3, 128.6, 128.5, 128.2, 128.0, 125.0, 124.2, 117.4, 116.4, 112.4, 72.0, 60.9, 21.3. LC-TOF/MS [M + H]^+^; Calculated for C_22_H_21_N_2_O: 329.1648, Found: 329.1834.

##### 3-((benzyloxy)methyl)−2-(4-methoxyphenyl)imidazo[1,2-a]pyridine (IPO8)

Brown solid, M.p.: 88–90 °C, Yield: 77%, Rf: 0.125 (ethyl acetate/n-hexane, 1/3). ^1^H NMR (400 MHz, CDCl_3_) δ 8.06 (d, J = 6.7 Hz, 1H, Ar–H), 7.73–7.55 (m, 3H, Ar–H), 7.35–7.17 (m, 6H, Ar–H), 6.98–6.90 (m, 2H, Ar–H), 6.78 (t, J = 6.7 Hz, 1H, Ar–H), 4.87 (s, 2H, -OCH_2_-), 4.55 (s, 2H, -OCH_2_-), 3.82 (s, 3H, -OCH_3_). ^13^C NMR (100 MHz, CDCl_3_) δ 159.5, 145.6, 145.2, 137.5, 129.8, 128.5, 128.2, 128.0, 126.6, 124.0, 124.1, 117.2, 116.1, 114.0, 112.3, 72.0, 60.9, 55.3. LC-TOF/MS [M + H]^+^; Calculated for C_22_H_21_N_2_O_2_: 345.1598, Found: 345.1793.

##### 3-((benzyloxy)methyl)−2-(4-ethoxyphenyl)imidazo[1,2-a]pyridine (IPO9)

Gray solid, M.p.: 109–110 °C, Yield: 86%, Rf: 0.125 (ethyl acetate/n-hexane, 1/3). ^1^H NMR (400 MHz, CDCl_3_) δ 8.07 (d, J = 6.6 Hz, 1H, Ar–H), 7.69–7.57 (m, 3H, Ar–H), 7.41–7.24 (m, 5H, Ar–H), 7.19 (t, J = 7.8 Hz, 1H, Ar–H), 6.99–6.88 (m, AA’BB’ system, 2H, Ar–H), 6.79 (t, J = 6.6 Hz, 1H, Ar–H), 4.88 (s, 2H, -OCH_2_-), 4.55 (s, 2H, -OCH_2_-), 4.06 (q, J = 6.8 Hz, 2H, -OCH_2_CH_3_), 1.42 (t, J = 6.8 Hz, 3H, -OCH_2_CH_3_). ^13^C NMR (100 MHz, CDCl_3_) δ 158.9, 145.6, 145.1, 137.5, 129.8, 128.5, 128.2, 128.0, 126.4, 125.0, 124.1, 117.2, 116.0, 114.5, 112.3, 72.0, 63.4, 60.9, 14.9. LC-TOF/MS [M + H]^+^; Calculated for C_23_H_23_N_2_O_2_: 359.1754, Found: 359.1995.

##### 3-((benzyloxy)methyl)−2-(4-(trifluoromethyl)phenyl)imidazo[1,2-a]pyridine (IPO10)

Orange solid, M.p.: 80–81 °C, Yield: 84%, Rf: 0.25 (ethyl acetate/n-hexane, 1/3). ^1^H NMR (400 MHz, CDCl_3_) δ 8.08 (d, J = 6.8 Hz, 1H, Ar–H), 7.73–7.71 (m, AA’BB’ system, 2H, Ar–H), 7.63 (d, J = 9.05 Hz, 1H, Ar–H), 7.41–7.31 (m, 5H, Ar–H), 7.25–7.21 (m, 3H, Ar–H), 6.82 (t, J = 6.8 Hz, 1H, Ar–H), 4.85 (s, 2H, -OCH_2_-), 4.56 (s, 2H, -OCH_2_-). ^13^C NMR (100 MHz, CDCl_3_) δ 149.9, 145.2, 144.3, 137.2, 132.7, 130.0, 128.6, 128.3, 128.2, 125.5, 124.3, 121.8, 121.0, 117.5, 116.9, 112.7, 72.1, 60.4. LC-TOF/MS [M + H]^+^; Calculated for C_21_H_19_N_2_O: 383.1366, Found: 383.1528.

##### 3-((benzyloxy)methyl)−2-(4-(trifluoromethoxy)phenyl)imidazo[1,2-a]pyridine (IPO11)

Brown viscous liquid, Yield: 81%, Rf: 0.225 (ethyl acetate/n-hexane, 1/3). ^1^H NMR (400 MHz, CDCl_3_) δ 8.09 (d, J = 6.8 Hz, 1H, Ar–H), 7.74–7.71 (m, AA’BB’ system, 2H, Ar–H), 7.63 (d, J = 9.1 Hz, 1H, Ar–H), 7.42–7.14 (m, 8H, Ar–H), 6.84 (t, J = 6.8 Hz, 1H, Ar–H), 4.86 (s, 2H, -OCH_2_-), 4.57 (s, 2H, -OCH_2_-), 3.82 (s, 3H, -OCH_3_). ^13^C NMR (100 MHz, CDCl_3_) δ 148.9, 145.3, 144.3, 137.2, 132.8, 129.9, 128.6, 128.3, 128.2, 125.4, 124.2, 121.0, 117.5, 116.9, 112.7, 72.1, 60.4. LC-TOF/MS [M + H]^+^; Calculated for C_22_H_18_F_3_N_2_O_2_: 399.1315, Found: 399.1542.

##### 3-((benzyloxy)methyl)−2-(4-nitrophenyl)imidazo[1,2-a]pyridine (IPO12)

Light red solid, M.p.: 113–115 °C, Yield: 77%, Rf: 0.225 (ethyl acetate/n-hexane, 1/3). ^1^H NMR (400 MHz, CDCl_3_) δ 8.23–8.21 (m, AA’BB’ system, 2H, Ar–H), 8.09 (d, J = 6.7 Hz, 1H, Ar–H), 7.88–7.86 (m, AA’BB’ system, 2H, Ar–H), 7.66 (d, J = 8.9 Hz, 1H, Ar–H), 7.36–7.27 (m, 6H, Ar–H), 6.89 (t, J = 6.7 Hz, 1H, Ar–H), 4.88 (s, 2H, -OCH_2_-), 4.62 (s, 2H, -OCH_2_-). ^13^C NMR (100 MHz, CDCl_3_) δ 147.2, 145.4, 142.9, 140.4, 137.0, 129.1, 128.7, 128.4, 128.3, 126.1, 124.3, 123.9, 118.2, 117.7, 113.2, 72.4, 60.2. LC-TOF/MS [M + H]^+^; Calculated for C_21_H_18_N_3_O_3_: 360.1343, Found: 360.1501.

##### 2-([1,1'-biphenyl]−4-yl)−3-((benzyloxy)methyl)imidazo[1,2-a]pyridine (IPO13)

Yellow solid, M.p.: 113–115 °C, Yield: 86%, Rf: 0.125 (ethyl acetate/n-hexane, 1/3). ^1^H NMR (400 MHz, CDCl_3_) δ 8.12 (d, J = 6.3 Hz, 1H, Ar–H), 7.84–7.77 (m, AA’BB’ system, 2H, Ar–H), 7.71–7.60 (m, 5H, Ar–H), 7.46 (t, J = 6.8 Hz, 2H, Ar–H), 7.38–7.21 (m, 7H, Ar–H), 6.85 (t, J = 6.3 Hz, 1H, Ar–H), 4.95 (s, 2H, -OCH_2_-), 4.59 (s, 2H, -OCH_2_-). ^13^C NMR (100 MHz, CDCl_3_) δ 145.2, 140.7, 137.4, 132.8, 129.0, 128.8, 128.5, 128.2, 128.1, 127.4, 127.3, 127.1, 125.3, 124.3, 117.4, 116.8, 112.5, 72.0, 60.8. LC-TOF/MS [M + H]^+^; Calculated for C_27_H_23_N_2_O: 391.1805, Found: 391.2053.

##### 3-((benzyloxy)methyl)−2-(naphthalen-2-yl)imidazo[1,2-a]pyridine (IPO14)

Gray viscous liquid, Yield: 80%, Rf: 0.15 (ethyl acetate/n-hexane, 1/3). ^1^H NMR (400 MHz, CDCl_3_) δ 8.21 (s, 1H, Ar–H), 8.13 (d, J = 6.7 Hz, 1H, Ar–H), 7.94–7.80 (m, 4H, Ar–H), 7.70 (d, J = 9.0 Hz, 1H, Ar–H), 7.53–7.44 (m, 2H, Ar–H), 7.38–7.16 (m, 6H, Ar–H), 6.83 (t, J = 6.7 Hz, 1H, Ar–H), 4.98 (s, 2H, -OCH_2_-), 4.59 (s, 2H, -OCH_2_-). ^13^C NMR (100 MHz, CDCl_3_) δ 145.6, 145.4, 137.5, 133.4, 133.0, 131.4, 128.5, 128.4, 128.2, 128.1, 128.0, 127.7, 127.6, 126.5, 126.2, 125.3, 124.3, 117.5, 117.0, 112.5, 72.1, 61.0. LC-TOF/MS [M + H]^+^; Calculated for C_25_H_21_N_2_O: 365.1648, Found: 365.1886.

### Cell culture and materials

Human cancer cell lines including MDA-MB-231 (human triple-negative breast adenocarcinoma, ATCC HTB-26), MCF-7 (human estrogen receptor-positive breast adenocarcinoma, ATCC HTB-22), HepG2 (human hepatocellular carcinoma, ATCC HB-8065), PC-3 (human prostate adenocarcinoma, ATCC CRL-1435), and A549 (human non-small cell lung carcinoma, ATCC CRM-CCL-185), together with the murine fibroblast cell line NIH/3T3 (mouse embryonic fibroblast, ATCC CRL-1658), were cultured in Dulbecco’s Modified Eagle Medium (DMEM; Thermo Fisher Scientific, USA) supplemented with 10% fetal bovine serum (FBS) and 1% penicillin–streptomycin. The non-tumorigenic human breast epithelial cell line MCF-10A (ATCC CRL-10317) was maintained in Mammary Epithelial Cell Growth Medium (Lonza/Clonetics, USA) supplemented with MEGM™ SingleQuots according to the manufacturer’s instructions. All cell lines were incubated at 37 °C in a humidified atmosphere containing 5% CO₂. The Human BCL-2 ELISA kit (ab119506) and the caspase-3 activity assay kit (ab39401) were obtained from Abcam (Cambridge, MA, USA). The MTT reagent, along with all other chemicals and reagents used in this study, was sourced from Sigma-Aldrich (St. Louis, MO, USA).

### MTT assay

Cell viability was assessed using an MTT-based colorimetric assay. Briefly, cells were seeded at 5 × 10^3^ cells per well in 96-well plates, allowed to attach, and then treated with the test compounds at concentrations ranging from 0 to 300 µM for 24 h. After exposure, MTT reagent (final concentration: 5 µg/mL) was added to each well, and the plate was incubated to allow formazan formation. The resulting crystals were subsequently solubilized in 100 µL dimethyl sulfoxide (DMSO), and absorbance was recorded at 540 nm using a microplate reader. Cells treated with 0.1% (v/v) DMSO served as the vehicle control, while Doxorubicin served as the positive control. Half-maximal inhibitory concentration (IC_50_) values were calculated from dose–response curves. The selectivity index (SI) was defined as the ratio of IC₅₀ values obtained in non-tumorigenic cells to those in cancer cells. For breast cancer cell lines, IC_50_ values from MCF-10A cells were used as the reference, whereas those from NIH/3T3 cells were used for the remaining cancer cell lines (Kuzu & Arzuk 2025).

### Assessment of BCL-2 and bax levels, mitochondrial membrane potential, and caspase-3 activity

BCL-2 and Bax protein levels, mitochondrial membrane potential, and caspase-3 activity were evaluated using commercially available assay kits. BCL-2 protein levels and caspase-3 activity were determined using kits obtained from Abcam (Cambridge, MA, USA) (ab119506 and ab39401, respectively). Bax protein levels and mitochondrial membrane potential were assessed using the Human Bax ELISA kit (E-EL-H0562) and the JC-1 assay kit (E-CK-A301), respectively, both purchased from Elabscience (Houston, TX, USA).

Cells were seeded into appropriate culture plates and treated with selected **IPO** derivatives at their respective IC_50_ concentrations for 24 h. Doxorubicin was included as a positive control in all mechanistic assays and was tested under the same experimental conditions. Following treatment, cells were harvested and lysed for protein extraction. Total protein concentrations were determined using the Bradford assay according to standard procedures. For BCL-2 and Bax analysis, equal amounts of protein were subjected to ELISA according to the manufacturer’s instructions, and absorbance was measured using a microplate reader. BCL-2 and Bax levels were expressed as ng/mg total protein. The Bax/BCL-2 ratio was subsequently calculated as an indicator of the balance between pro-apoptotic and anti-apoptotic signaling.

Caspase-3 activity was determined using a colorimetric assay based on the cleavage of a specific peptide substrate and expressed as fold change relative to untreated control cells. Mitochondrial membrane potential was evaluated using the JC-1 fluorescent probe according to the manufacturer's protocol. Red and green fluorescence intensities were measured using a fluorescence microplate reader at the recommended wavelengths. Results were expressed as the JC-1 red-to-green fluorescence ratio (% of untreated control).

### Statistical analysis

All data are presented as mean ± standard deviation (SD). Experiments were performed in triplicate and repeated at least three independent times. IC_50_ values were calculated using GraphPad Prism® version 8 (GraphPad Software, San Diego, CA, USA) based on nonlinear regression analysis of dose–response curves. Statistical comparisons between groups were performed using one-way analysis of variance (ANOVA) followed by appropriate post hoc tests. A p-value of < 0.05 was considered statistically significant.

### Molecular docking studies

Molecular docking studies were conducted using AutoDock 4.2 software to identify the interactions of compounds (**IPO8, IPO9, IPO10, IPO11,** and **IPO13**) against 3D structures of BCL-2. The crystal structure of the BCL-2 (PDB ID: 4MAN) (Souers et al. 2013) was obtained from the RCSB Protein Data Bank (www.rcsb.org). The molecular structures of the compounds were drawn using GaussView 5.0 and then optimized using DFT with the Gaussian 03 package at the B3LYP/6-31G level. Docking processes started with removing the unwanted solvent, water, and ligands from the BCL-2 structure. In docking studies, the coordinate centers were determined as BCL-2 x: −12.621; y: 8.675; z: 7.516. Then, using a 50 × 50 × 50 grid centered on the predicted locations and a grid spacing of 0.375 Å, the lowest-energy conformations were selected for further study. Water molecules were removed using AutoDock Tools, and polar hydrogen atoms, Gasteiger partial charges, and Kollman charges were subsequently added to the targets. Additionally, the rotatable bonds of the compounds were adjusted. The target regions of the selected compounds were docked under validated conditions using AutoDock 4.2. To validate the docking process for the specified target, the native ligand was redocked and overlapped with the experimentally determined crystal structure. The Lamarckian genetic algorithm (10 runs) approach was applied, with a population size of 300 in both simulations. The interactions of the crystal structure of BCL-2 with the compounds were analyzed using the Discovery Studio Client 4.1 program.

### Pre-ADMET calculations

The ADMET properties of the compounds were determined by online web tools. The SMILE of each compound was obtained from SwissADME (https:/www.swissadme.ch). The absorption, distribution, excretion, metabolism (CYP450 substrate or inhibitor), and toxicity parameters were evaluated by ADMETLab 2.0 (Xiong et al 2021).

## Supplementary Information

Below is the link to the electronic supplementary material.

Supplementary file1 (DOC 3615 KB)

## Data Availability

All figures and schemes presented in this article are original and were generated by the authors during the study. The supporting spectral data (including NMR and LC-TOF spectra) for all synthesized molecules are available in the Supplementary Information file submitted with this article. No external datasets, software, or code were used or generated in this study.
