# Supplementary material for: Synthesis and biological evaluation of benzyloxymethyl-substituted imidazo[1,2-a]pyridines as apoptosis-inducing anticancer agents through modulation of BCL-2/Bax signaling and caspase-3 activation
Source: Naunyn Schmiedebergs Arch Pharmacol. 2026 Jun 26;399(12):19537–54. doi: 10.1007/s00210-026-05638-6 (PMC13593677; doi:10.1007/s00210-026-05638-6)
Supplement: Supplementary file 1 [file 210_2026_5638_MOESM1_ESM.doc]

**Synthesis and Biological Evaluation of Benzyloxymethyl-Substituted Imidazo[1,2-a]pyridines as Apoptosis-Inducing Anticancer Agents through Modulation of BCL-2/Bax Signaling and Caspase-3 Activation**

Ege ARZUK^1^, İbrahim PINAR^2^, Burak KUZU^2,^*

^1^Department of Pharmaceutical Toxicology, Faculty of Pharmacy, Ege University, İzmir, 35040, Türkiye

^2^Department of Pharmaceutical Chemistry, Faculty of Pharmacy, Van Yuzuncu Yil University, Van, 65080, Türkiye

**Corresponding author:*

Dr. Burak KUZU, Department of Pharmaceutical Chemistry, Faculty of Pharmacy, Van Yuzuncu Yil University, Van, 65080, Türkiye; [burakkuzu@yyu.edu.tr](mailto:burakkuzu@yyu.edu.tr)

**Supplementary Material**

| **Contents** | **Pages** |
| --- | --- |
| 1. Structures of the synthesized compounds **IPO1-14** | **2** |
| 2. ^1^H- and ^13^C-NMR spectrum copies of compounds **IPO1-14** | **3** |
| 3. LC-TOF/MS [M+H]^+^ spectrum copies of compounds **IPO1-14** | **17** |

1. Structures of the synthesized compounds **IPO1-14**

2. ^1^H- and ^13^C-NMR spectrum copies of compounds **IPO1-14**

**Figure S1.** ^1^H-NMR spectrum of **iPO-1**

**Figure S2.** ^13^C-NMR spectrum of **iPO-1**

**Figure S3.** ^1^H-NMR spectrum of **iPO-2**

**Figure S4.** ^13^C-NMR spectrum of **iPO-2**

**Figure S5.** ^1^H-NMR spectrum of **iPO-3**

**Figure S6.** ^13^C-NMR spectrum of **iPO-3**

**Figure S7.** ^1^H-NMR spectrum of **iPO-4**

**Figure S8.** ^13^C-NMR spectrum of **iPO-4**

**Figure S9.** ^1^H-NMR spectrum of **iPO-5**

**Figure S10.** ^13^C-NMR spectrum of **iPO-5**

**Figure S11.** ^1^H-NMR spectrum of **iPO-6**

**Figure S12.** ^13^C-NMR spectrum of **iPO-6**

**Figure S13.** ^1^H-NMR spectrum of **iPO-7**

**Figure S14.** ^13^C-NMR spectrum of **iPO-7**

**Figure S15.** ^1^H-NMR spectrum of **iPO-8**

**Figure S16.** ^13^C-NMR spectrum of **iPO-8**

**Figure S17.** ^1^H-NMR spectrum of **iPO-9**

**Figure S18.** ^13^C-NMR spectrum of **iPO-9**

**Figure S19.** ^13^C-NMR spectrum of **iPO-10**

**Figure S20.** ^13^C-NMR spectrum of **iPO-10**

**Figure S21.** ^1^H-NMR spectrum of **iPO-11**

**Figure S22.** ^13^C-NMR spectrum of **iPO-11**

**Figure S23.** ^1^H-NMR spectrum of **iPO-12**

**Figure S24.** ^13^C-NMR spectrum of **iPO-12**

**Figure S25.** ^1^H-NMR spectrum of **iPO-13**

**Figure S26.** ^13^C-NMR spectrum of **iPO-13**

**Figure S25.** ^1^H-NMR spectrum of **iPO-14**

**Figure S28.** ^13^C-NMR spectrum of **iPO-14**

3. LC-TOF/MS [M+H]+ Low and Range spectrum copies of compounds **IPO1-14**

**Figure S29**: LC-TOF/MS [M+H]^+^ spectrum of compound **IPO-1**

**Figure S30**: LC-TOF/MS [M+H]^+^ spectrum of compound **IPO-2**

**Figure S31**: LC-TOF/MS [M+H]^+^ spectrum of compound **IPO-3**

**Figure S32**: LC-TOF/MS [M+H]^+^ spectrum of compound **IPO-4**

**Figure S33**: LC-TOF/MS [M+H]^+^ spectrum of compound **IPO-5**

**Figure S34**: LC-TOF/MS [M+H]^+^ spectrum of compound **IPO-6**

**Figure S45**: LC-TOF/MS [M+H]^+^ spectrum of compound **IPO-7**

**Figure S36**: LC-TOF/MS [M+H]^+^ spectrum of compound **IPO-8**

**Figure S37**: LC-TOF/MS [M+H]^+^ spectrum of compound **IPO-9**

**Figure S38**: LC-TOF/MS [M+H]^+^ spectrum of compound **IPO-10**

**Figure S39**: LC-TOF/MS [M+H]^+^ spectrum of compound **IPO-11**

**Figure S40**: LC-TOF/MS [M+H]^+^ spectrum of compound **IPO-12**

**Figure S41**: LC-TOF/MS [M+H]^+^ spectrum of compound **IPO-13**

**Figure S42**: LC-TOF/MS [M+H]^+^ spectrum of compound **IPO-14**
